# Filament formation and NAD processing by noncanonical human FAM118 sirtuins

**DOI:** 10.1038/s41594-025-01715-1

**Published:** 2025-11-17

**Authors:** Domagoj Baretić, Sophia Missoury, Karishma Patel, Maximilien Martinez, Franck Coste, Kang Zhu, Rebecca Smith, Anna Georgina Kopasz, Yang Lu, Nicolas Bigot, Catherine Chapuis, Romane Riou, Nina Đukić, Stéphane Goffinont, Valentin Pressoir, Sara Patačko, Gyula Timinszky, Marc Delarue, Bertrand Castaing, Dragana Ahel, Andreja Mikoč, Sébastien Huet, Ivan Ahel, Marcin J. Suskiewicz

**Affiliations:** 1 Sir William Dunn School of Pathology, University of Oxford, Oxford, UK; 2 Architecture and Dynamics of Biological Macromolecules, Institut Pasteur, UMR 3528, Université Paris Cité, CNRS, Paris, France; 3 IGDR (Institut de Génétique et Développement de Rennes), UMR 6290 & BIOSIT (Biologie, Santé, Innovation Technologique), UAR 3480, Université Rennes, CNRS, Rennes, France; 4 Centre de Biophysique Moléculaire (CBM), UPR 4301, CNRS, Orléans, France; 5 Laboratory of DNA Damage and Nuclear Dynamics, Institute of Genetics, HUN-REN Biological Research Centre, Szeged, Hungary; 6 Doctoral School of Multidisciplinary Medical Sciences, University of Szeged, Szeged, Hungary; 7 Division of Molecular Biology, Ruđer Bošković Institute, Zagreb, Croatia

**Keywords:** Nucleotide-binding proteins, Cryoelectron microscopy, Transferases, Cellular imaging, Supramolecular assembly

## Abstract

Sirtuins are an ancient family of enzymes with diverse nicotinamide adenine dinucleotide (NAD)-dependent activities. Here we identify family with sequence similarity 118 member B (FAM118B) and FAM118A—two understudied vertebrate proteins—as vertebrate-specific sirtuins with similarities to bacterial antiphage sirtuins. We show that human FAM118B forms head-to-tail filaments both in vitro and in living human cells, a feature that appears to be conserved in both FAM118B and its paralog FAM118A across vertebrates. While human FAM118B and FAM118A have individually very weak NAD-processing activity in vitro, their interaction leads to markedly increased activity, suggesting a tightly regulated system. The overexpression of wild-type human FAM118B and FAM118A leads to strongly decreased NAD levels in human cells, an effect that is abolished in catalytically dead or filament-deficient mutants. Our study highlights filament formation and NAD processing as conserved mechanisms among immunity-associated sirtuins across evolution.

## Abstract (web summary)

Baretić and Missoury et al. identify vertebrate proteins FAM118B and FAM118A as sirtuins similar to bacterial antiphage enzymes and show that FAM118A/B processing of NAD involves head-to-tail filament formation and a partnership between the two paralogs.

## Main

The sirtuin protein family gained prominence through studies on human SIRT proteins, key epigenetic and metabolic regulators^1^ with severe knockout phenotypes in mice^2–4^. However, under its original definition, ‘sirtuin’ (Sir2-like protein) refers broadly to all homologs of the yeast epigenetic factor silent information regulator 2 (Sir2)^5^ across the tree of life, from bacteria to humans^6–9^, and additional family members are still being identified and characterized.

Structurally, sirtuins are defined by a catalytic domain with a large Rossmann-fold subdomain and a small subdomain that features an α-helical module and, in some family members, a zinc-binding β-sheet module^10,11^. Catalytically, sirtuins generally process the ubiquitous cellular cofactor nicotinamide adenine dinucleotide (NAD), cleaving the nicotinamide group and transferring the remaining adenosine diphosphate (ADP)-ribosyl moiety onto a nucleophilic acceptor^12^. However, the nature of this acceptor varies among sirtuins, leading to various specific activities.

Protein deacetylation is believed to be the primary function of human SIRTs and yeast Sir2. These enzymes transfer the ADP-ribosyl moiety from NAD onto acetyl groups on protein lysine residues, provoking the subsequent release of acetyl as 2′-*O*-acetyl-ADP-ribose^8,13–15^. 2′-*O*-Acetyl-ADP-ribose can potentially serve as a secondary messenger^16^ but is usually metabolized into ADP-ribose and acetate^17–19^. Some sirtuins use the same NAD-dependent mechanism to remove other acyl modifications such as succinyl, malonyl or fatty acyl groups^20,21^.

Another occasionally reported sirtuin activity is protein ADP-ribosylation, a post-translational modification (PTM) more commonly associated with the poly(ADP-ribose) polymerase (PARP) enzyme family^22^. Here, the ADP-ribosyl moiety originating from NAD becomes covalently attached to an amino acid residue on a substrate protein. Protein ADP-ribosylation is the primary function of macrodomain-linked sirtuins (SirTMs) found in fungi and bacteria^23^ and might be at least a secondary function of human SIRT4 and SIRT6 (refs. ^24,25^).

Lastly, recent research suggests that certain sirtuins work primarily as NAD glycohydrolases (NADases), by transferring the ADP-ribosyl moiety from NAD onto a water molecule. This function has been proposed mainly for the recently characterized bacterial sirtuins implicated in antiphage immunity^7,26–33^. Given NAD’s essential role, its hydrolysis provides a mechanism for inducing growth arrest or cell death in phage-infected cells, thereby limiting infection spread^7,29,34^. The activation of antiphage sirtuins often involves a change in their multimerization state, including cases of filament formation^32,35–38^.

To address a gap in sirtuin characterization, this study examines two poorly characterized vertebrate sirtuins: family with sequence similarity 118 member B (FAM118B) and its paralog FAM118A. Our phylogenetic analysis, consistent with recent work^39^, indicates that they are closer to bacterial antiphage sirtuins than to human SIRTs. We further show that, like some of its bacterial homologs, human FAM118B forms filaments in vitro and in cells, but with a distinct head-to-tail topology, suggesting independent evolution of filamentation. We also demonstrate that, while human FAM118B or FAM118A individually have minimal NAD-processing activity in vitro, their combination results in markedly higher NAD-processing activity. Lastly, their coexpression in cells strongly depletes cellular NAD levels, validating a previously unrecognized eukaryotic NAD-processing system.

## Results

### Human FAM118s are similar to bacterial antiphage sirtuins

We encountered human FAM118B and FAM118A when searching for uncharacterized sirtuins in InterPro and Pfam databases, where they are both annotated with a SIR2-like (SIR2_2) domain. Analyzing them in 2019 with HHPred^40^, which searches the Protein Data Bank (PDB) for entries with sequence and secondary-structure similarity, confirmed that both FAM118s are significantly similar to human SIRT1 (PDB 4KXQ, *E* values of 2.9 × 10^−12^ for FAM118B and 5.3 × 10^−^^13^ for FAM118A).

Repeating the HHPred analysis in 2022, after several bacterial sirtuin structures were deposited to the PDB, predicted a stronger similarity between FAM118s and these bacterial enzymes than with canonical sirtuins. Notably, ThsA from *Streptococcus* *equi* ranked as the strongest hit for both FAM118B and FAM118A (PDB 7UXT, *E* values of 1.2 × 10^−25^ for FAM118B and 2.3 × 10^−26^ for FAM118A). At the time of this article’s submission, an even better score was observed for *Escherichia* *coli* ThsA (PDB 8WC0, *E* values of 9.6 × 10^−26^ for FAM118B and 1.3 × 10^−26^ for FAM118A).

**Fig. 1:**
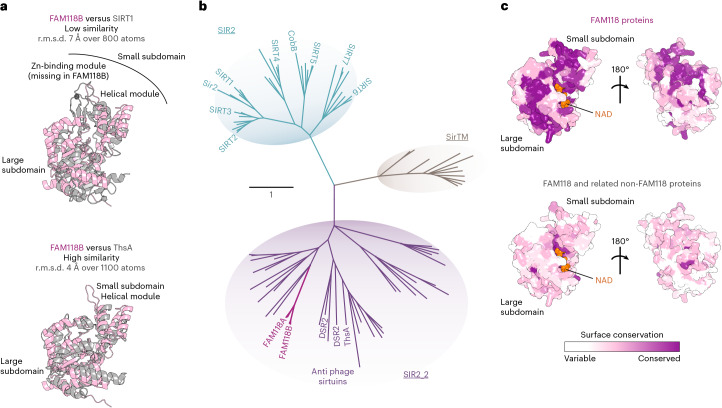
FAM118s are a distinct clade of noncanonical sirtuins. **a**, Structural superposition of AF3 model of human FAM118B (Extended Data Fig. 1a) and crystallography-derived models of sirtuin domains of human SIRT1 (PDB 4KXQ, top) or bacterial ThsA (PDB 7UXT, bottom). The approximate root mean square deviation (r.m.s.d.) for a specified number of well-aligned atoms is provided. **b**, Phylogenetic tree of sirtuin protein family drawn to scale, with branch lengths reflecting the number of substitutions per site. A more detailed version is provided in Extended Data Fig. 2. The following subfamilies are distinguished (names underlined): SIR2, SIR2_2 and SirTM. The names of selected proteins are indicated. **c**, AF3 model of human FAM118B (only residues 28–325 are shown) bound to NAD (orange) (model details in Extended Data Fig. 1c) with protein surface colored according to sequence conservation among either a representative set of FAM118 SIR_2 proteins (top) or a set including both FAM118 and related non-FAM118 SIR2_2 proteins (bottom). The hydrophobic core (not shown) and NAD-binding site are conserved in both cases, while the FAM118 clade also shares conserved surface patches. Left, views in the same orientation as FAM118B in **a**.

**Extended Data Fig. 1:**
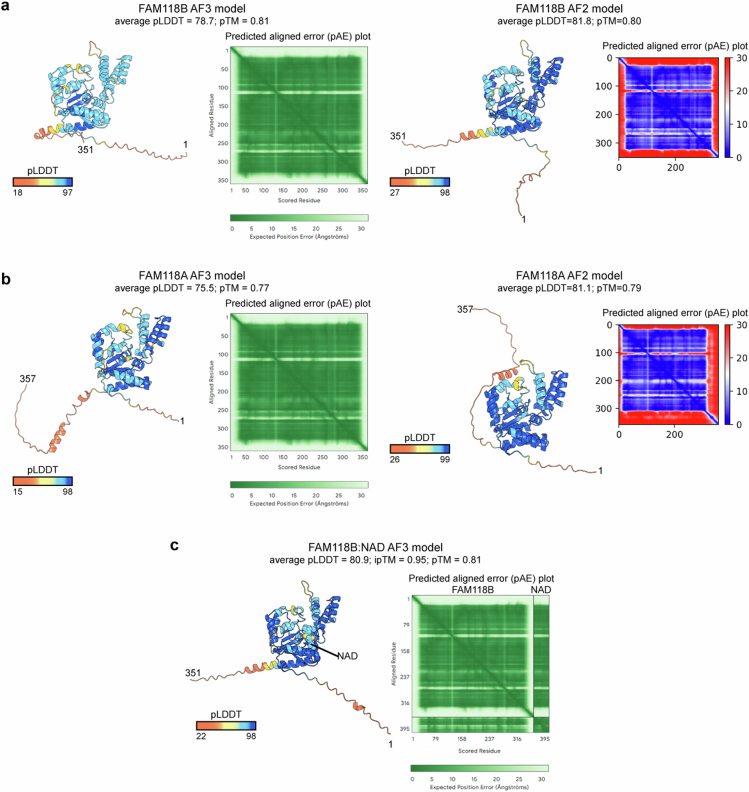
AlphaFold modeling of FAM118 monomers and FAM118B:NAD complex. AlphaFold 3 (AlphaFold server) or AlphaFold 2 (ColabFold v1.5.5) models are provided as indicated with AF3 or AF2, respectively. For each protein or complex the top model out of five calculated models is shown, coloured according to the per-residue predicted local distance difference test (pLDDT) score. First and last residues in a chain are numbered. The range of per-residue pLDDT values for each model is indicated on a scale provided. The accompanying average pLDDT score, interface predicted template modeling (ipTM) score (for complexes only), and predicted template modeling (pTM) score are also provided, together with a predicted aligned error (pAE) plot, coloured according to the indicated scales. **a**, Human FAM118B monomer models obtained using AF3 (left) and AF2 (right). The AF3 model is used in Figs. 1a, 2g, and 3f. **b**, Human FAM118A monomer models obtained using AF3 (left) and AF2 (right). **c**, AF3 model of human FAM118B in complex with NAD (indicated). The model was used in Figs. 1c, 7a, and Supplementary Figs. 1 and 2.

With the advent of AlphaFold (AF), we generated structural models of both FAM118s using AF2 and AF3 and superposed them with structures of either human SIRT1 or *S*. *equi* ThsA, demonstrating a higher similarity of both FAM118s with ThsA compared to SIRT1 (Extended Data Fig. 1a,b and Fig. 1a). Notably, FAM118s and ThsA share a seven-stranded central β sheet in their large subdomain—contrasting with six β-strands in SIRT1—and both lack the zinc-binding module present in SIRT1.

### FAM118s belong to diverse SIR2_2 subfamily of sirtuins

**Extended Data Fig. 2:**
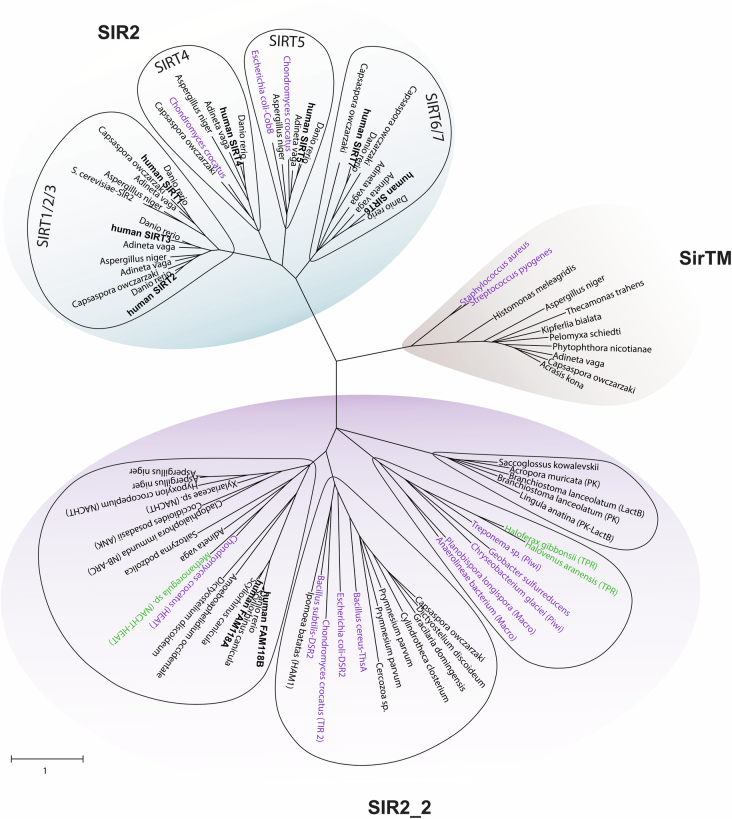
Phylogenetic tree of the sirtuin protein family. The maximum-likelihood tree shown here is identical to that in Fig. 1b, but presented differently. All sirtuin proteins included in the analysis are labelled along with their species of origin. For members of the SIRT2_2 subfamily, some species names are followed in brackets by the name or acronym of an additional protein domain present in the same protein (for example, Piwi, Macro, HEAT, TIR, TPR, *etc*.). Eukaryotic proteins are indicated in black, bacterial in purple, and archaeal in green. The tree is drawn to scale, with the branch lengths measured in the number of substitutions per site. SIR2, canonical sirtuins; SirTM, macrodomain-linked sirtuins; SIR2_2, SIR2-like. Source data

To further explore the relationship between FAM118s and other sirtuins, we constructed an evolutionary tree of representative sirtuin sequences (Fig. 1b and Extended Data Fig. 2). Beyond the canonical sirtuins (the SIR2 subfamily)—found primarily in eukaryotes with few bacterial representatives—the tree features two further distinct subfamilies. One is SirTM, whose members, found mainly in fungi and bacteria, catalyze protein ADP-ribosylation, as we demonstrated previously^23,41^. The other, a much broader group, corresponds to the SIR2_2 category used by InterPro and Pfam (the name we adopt here for this subfamily) and to the recently proposed class of SIRims (SIR and immunity)^39^. The SIR2_2 subfamily consists of proteins from bacteria, archaea and eukaryotes, including human FAM118s alongside bacterial sirtuins ThsA^7,27,28,32^, DSR1 and DSR2 (refs. ^29,37^).

### FAM118s form a distinct vertebrate-specific clade within SIR2_2 subfamily

We next investigated how closely FAM118s resemble other SIR2_2 members. Sequence-based searches for FAM118B and FAM118A homologs revealed two sets of SIR2_2 proteins: a vertebrate-specific set with high sequence identity to human FAM118s (≥41%), termed henceforth the FAM118 clade, and a set of proteins with lower identity (<32%, and often <25%) found in a broader set of species including prokaryotes and corresponding to non-FAM118 SIR_2 proteins.

Mapping the amino acid residues conserved within the FAM118B clade onto an AF3 model of human FAM118B revealed conservation not only within the hydrophobic domain core and predicted NAD-binding site, expected for proteins sharing the same overall fold and NAD-binding capability, but also across extensive surface patches (Fig. 1c, top). The latter may imply shared homomultimerization or interaction properties. In contrast, when mapping, onto the same model, the residues conserved between FAM118s and the closest among non-FAM118 SIR2_2 proteins, we observed conservation only within the hydrophobic core and the predicted NAD-binding site (Fig. 1c, bottom).

### FAM118s can be grouped into FAM118B-type and FAM118A-type proteins

**Extended Data Fig. 3:**
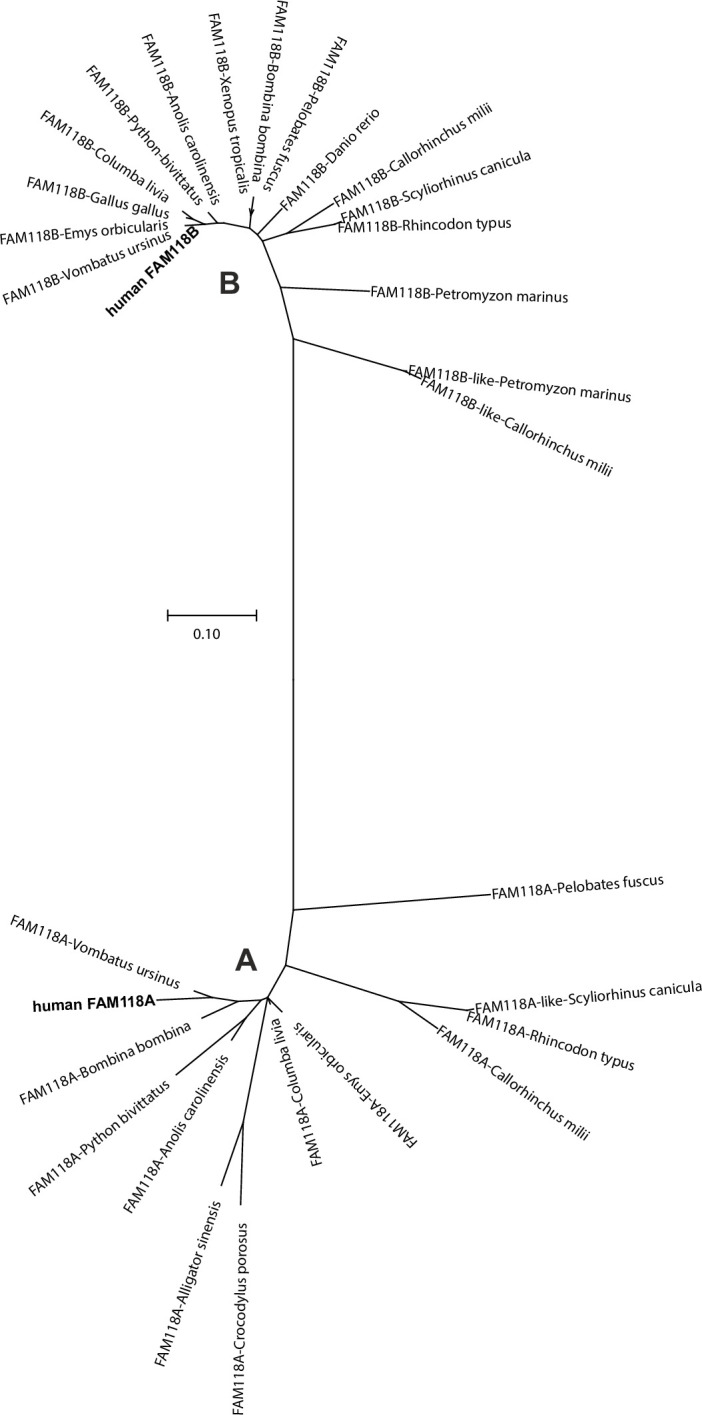
Phylogenetic tree of the FAM118 clade. The tree includes representative FAM118B and FAM118A proteins from various organisms from fish to humans. The tree is drawn to scale, with the branch lengths measured in the number of substitutions per site. All analysed proteins can be classified with high confidence as either FAM118A or FAM118B—with these two clusters designated as A or B—based on their higher similarity to human FAM118A or human FAM118B, respectively. Source data

A further phylogenetic analysis showed that, within the FAM118 clade, all proteins can be distinctly categorized into one of two clusters, corresponding to FAM118B-type or FAM118A-type proteins (Extended Data Fig. 3).

Looking specifically at the FAM118B orthologs, we observed that they are present in almost all vertebrates, from jawless fish to humans (with a few exceptions, notably in the genera *Myxine*, *Ascaphus*, *Alectura* and *Opisthocomus*), demonstrating the emergence of FAM118B before the common ancestor of all or most extant vertebrates. The FAM118A orthologs, on the other hand, are consistently missing from jawless fish, suggesting that FAM118A emerged by duplication of FAM118B after the separation of jawless and jawed fish lineages. Moreover, FAM118A proteins have apparently been lost in a number of other fishes and amphibians (with exceptions such as the catshark *Scyliorhinus* *canicula* or the toad *Pelobates* *fuscus*) and are more consistently retained among reptiles, birds and mammals, where an FAM118B and FAM118A pair is a norm. Rare exceptions that feature FAM118A but not FAM118B include newts, guan and tinamou birds and possibly alligators, crocodiles and gavials, which have low-quality FAM118A sequences.

### Recombinant human FAM118B forms filaments revealed by cryo-electron microscopy (cryoEM)

We set out to biochemically and structurally characterize a representative member of the FAM118 clade, focusing on human FAM118B. We recombinantly produced and purified full-length FAM118B (referred to simply as FAM118B) and its slightly truncated variant lacking poorly conserved and likely flexible terminal regions (designated FAM118B_24__–334_), both with a noncleavable His_6_ tag.

To elucidate FAM118B structure, we first attempted crystallography. Although both constructs crystallized, they did not diffract. In light of the insights below, this may be because of the difficulty of achieving orderly crystal packing from large multimeric units^42^.

**Fig. 2:**
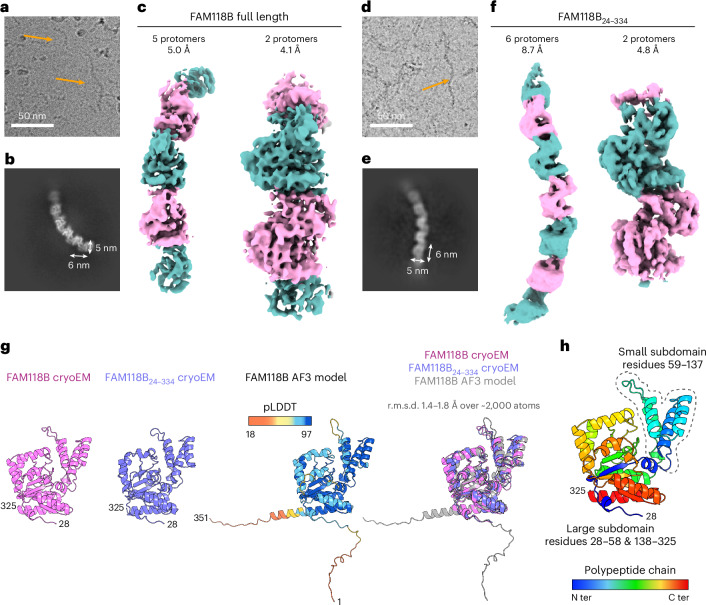
CryoEM analysis of human FAM118. **a**, A fragment of a representative cryoEM micrograph of full-length human FAM118B from *n* = 11,630 collected images. Two sample filaments are indicated with orange arrows. **b**, A sample two-dimensional class of FAM118B showing a filament fragment. **c**, CryoEM maps of FAM118B filament fragments contoured at 0.13 in ChimeraX, with alternating protomers colored pink and teal. The map that encompasses two full protomers additionally includes fragments of neighboring subunits. **d**,**e**, Same as in **a**,**b** but for an FAM118B variant lacking N and C extremities (FAM118B_24__–334_). The cryoEM micrograph is representative of *n* = 100 collected images. **f**, CryoEM map of FAM118B_24__–334_ filament fragments contoured at 0.2 in ChimeraX. **g**, Structural comparison of a representative protomer from the highest-resolution FAM118B and FAM118B_24__–334_ cryoEM models (only residues 28–325 are visible in electron density for both constructs) and an AF3 model of human FAM118B (from Extended Data Fig. 1a), each separately and all three superposed. The AF3 model is colored according to the provided pLDDT scale. The approximate r.m.s.d. for the specified number of well-aligned atoms is provided. **h**, CryoEM model of a FAM118B protomer (same as in **g**, left) in rainbow coloring from the N terminus (ter) to C terminus. The small subdomain is encircled with a dotted line.

**Extended Data Fig. 4:**
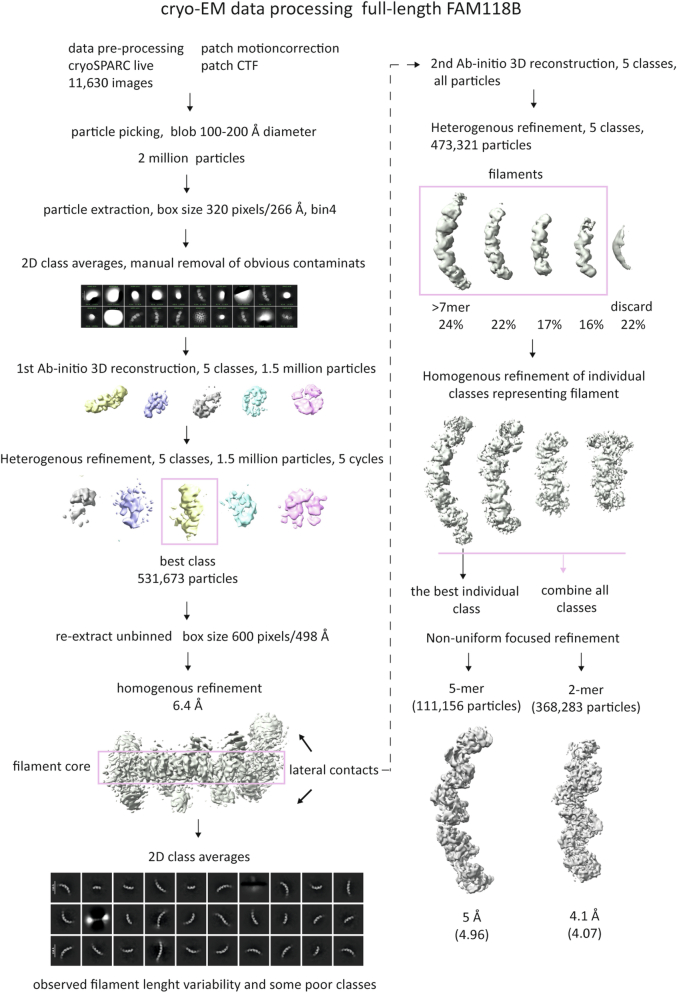
CryoEM analysis workflow for full-length human FAM118B. A cryoEM single dataset collected on Titan Krios 300 kV was processed in entirety using cryoSPARC and individual structure calculation steps are depicted in sequence with final refined and sharpened cryoEM maps shown for 2-protomer and 5-protomer fragments at the end of pipeline. Gold-standard Fourier shell correlation (FSC) 0.143 global resolution estimates are shown in Angstroms (Å) for each map.

**Extended Data Fig. 5:**
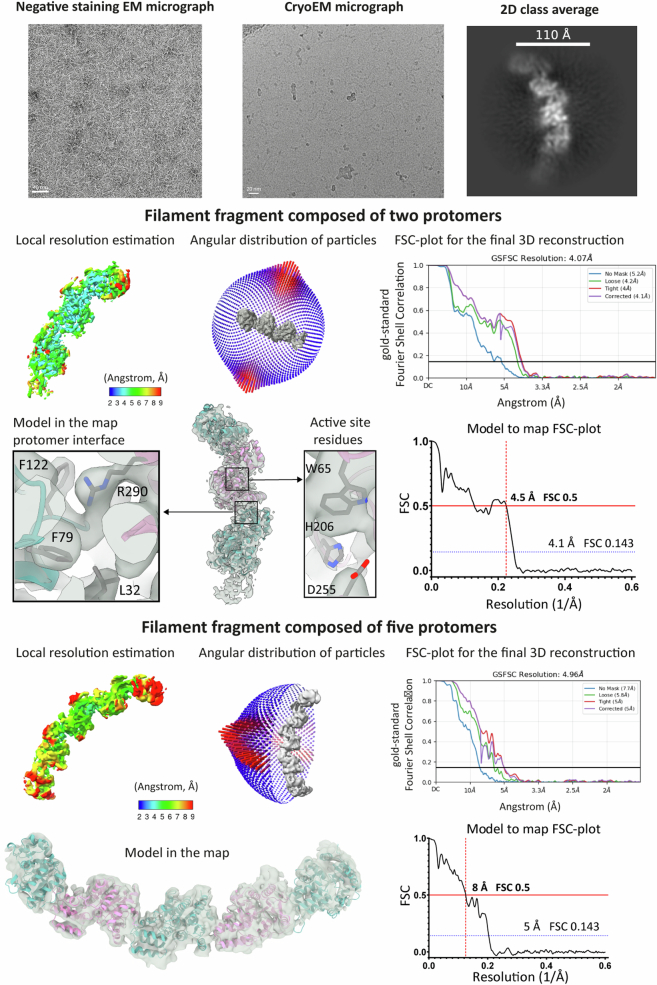
CryoEM data quality analysis for full-length human FAM118B. Negative-stain images (representative out of *n* = 20 similar images, scale bar 40 nm) and cryoEM images (representative out of *n* = 11,630 images, scale bar 20 nm) show predominantly single stranded filaments of varying lengths, but occasional sideway contacts between individual chains can be discerned. In a cryoEM 2D class average, secondary structures of several protomers forming a single filament can be observed (box size of 320 pixels was used for data processing in cryoSPARC^93^). Final refinement cryoEM maps, atomic models and data processing statistics are shown for 2-protomer and 5-protomer fragment of filaments. Calculation of local resolution^94^ was performed in cryoSPARC and the maps were rendered in ChimeraX^95^ by colour coding resolution according to the scale provided. 3D-representation of an angular distribution was originally calculated in cryoSPARC and data were converted using Pyem (DOI:10.5281/zenodo.3576629) and RELION^96^ for display in ChimeraX. Fourier shell correlation (FSC)-plots showing global resolution estimates for final refined cryoEM maps were generated in cryoSPARC. Higher resolution map and a corresponding model of a complete 2-protomer fragment of filament shows placement of bulky amino acid side chains in the density either at the interface between two protomers or within the active site. An overall map at lower resolution and a refined model are shown for 5-protomer fragment. Masked model to map FSC curves were calculated using EMAN2^97^ for both fragments. Masks were generated in cryoSPARC (soft padded with 25 pixels for 2-protomer or with 30 pixels for 5-protomer map) from unsharpened final cryoEM maps (sum of half-maps) pre-segmented in ChimeraX to exclude parts of the map not accounted for by each of the two models. FSC-plots were generated in GraphPad Prism 10 (www.graphpad.com) and map to model resolution estimates in Å were determined at a standard FSC 0.5 marked by crossed red lines.

**Extended Data Fig. 6:**
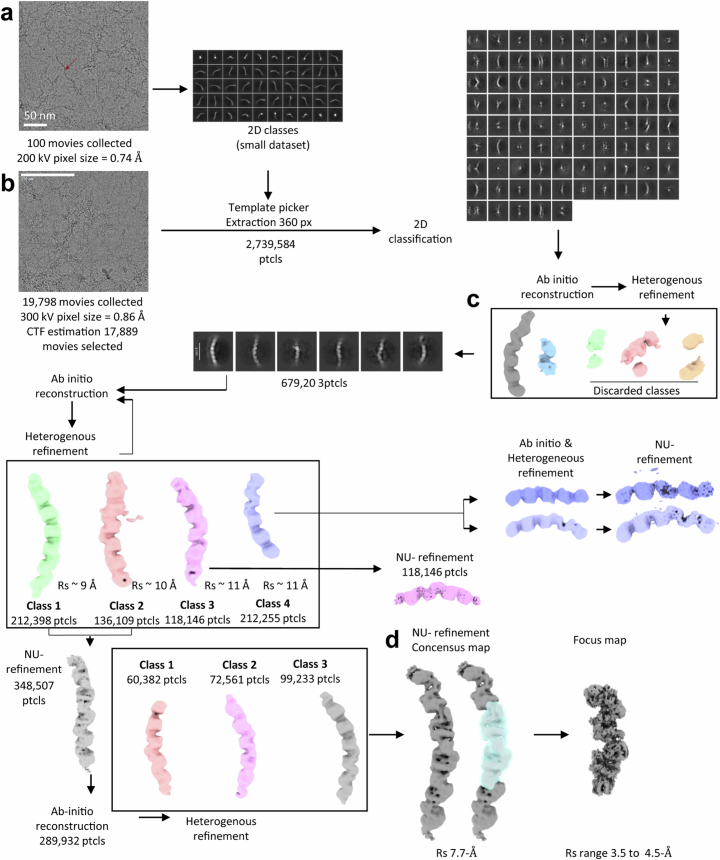
CryoEM analysis workflow for human FAM118B24-334. Rs, resolution; ptcls, particles. **a**, Representative cryoEM micrograph from a small dataset of *n* = 100 images collected on a 200-kV Glacios microscope, showing the FAM118B filament (indicated by red arrow). 2D classification was performed on this dataset and used as a template for processing the larger dataset. **b**, Representative cryoEM micrograph from a large dataset of *n* = 19,798 images collected on a 300-kV Titan Krios microscope, showing truncated versions of FAM118B filaments. Scale bar: 150 nm. Representative 2D class averages are shown. Particles from the best-resolved 2D classes were selected for further processing. **c**, Workflow of 3D reconstruction. Refinements were progressively improved through multiple rounds of 2D classification, ab initio reconstruction, and heterogeneous refinement, leading to the selection of the best-aligned particles. **d**, Non-uniform refinement was applied to the final set of particles. The filament did not exhibit a fixed symmetry and reached a global resolution of 7.5 Å. To improve map quality, a focused mask was applied on two monomers of FAM118B, yielding a final local refinement at 4.5 Å resolution. Fourier shell correlation (FSC) plot of each refinement is given in Extended Data Fig. 7.

**Extended Data Fig. 7:**
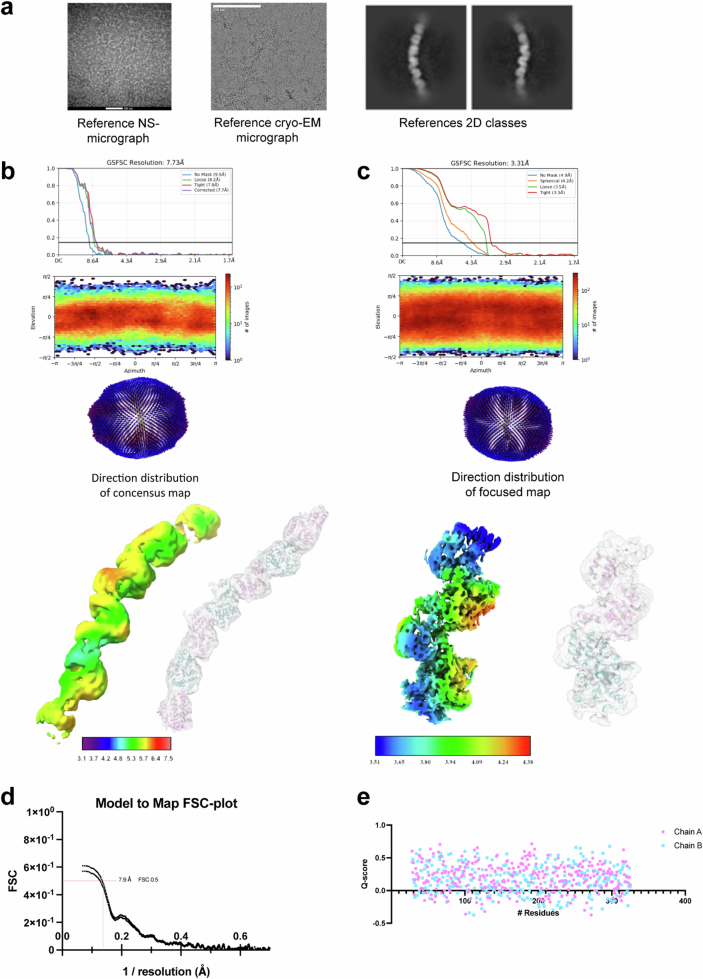
CryoEM data quality analysis for human FAM118B_24-334_. **a**, Representative negative-stain EM (out of *n* = 4 repeats, scale bar 100 nm) and cryoEM (out of *n* = 100 images, scale bar 150 nm) micrographs and corresponding 2D class averages of FAM118B filaments. **b**, Final cryoEM consensus map refined from a 6-protomers segment. **c**, Focused refinement map of a 2-protomers segment. Fourier shell correlation (FSC) plots for each refinement were generated using CryoSPARC^93^. Angular distribution plots of both the consensus and focused maps were also produced in CryoSPARC. Local resolution estimation was carried out using the Phenix suite^98^ and visualised in ChimeraX^95^.The focused map achieved higher resolution than the consensus map and was therefore used for model building and refinement of the 2-protomer segment. Model refinement was performed iteratively through manual adjustment in Coot^99^ and automated refinement in Phenix. **d**, The map-to-model FSC plot of 2-protomers model from the final Phenix refinement iteration is shown, with bars indicating resolution at FSC thresholds of 0.5. **e**, Q-score of 2-protomers model was calculated in ChimeraX. In blue and pink, the Q-score corresponding to chain A and chain B respectively.

We next turned to negative-stain transmission EM, where both FAM118B and FAM118B_24__–334_ manifested as filament-like objects of varying length (Extended Data Figs. 5 and 7). Subsequent cryoEM analysis confirmed the presence of filaments in both samples. To perform cryoEM reconstruction, we treated filament fragments composed of several protomers as a single particle (Fig. 2a–f, Extended Data Figs. 4–7 and Table 1). This approach produced electron density maps of FAM118B filament fragments composed of five and two protomers, at 5.0-Å and 4.1-Å resolution, respectively (both obtained using a focused refinement in the last step), and of FAM118B_24__–334_ filament fragments composed of six protomers at 7.8-Å resolution (without focused refinement) and two protomers at 4.8-Å resolution (with focused refinement). Despite the apparent helical symmetry of the filaments, an asymmetric reconstruction approach produced higher-resolution densities than helical reconstruction, similar to what was observed for another filamentous systems^43^, likely because of structural heterogeneity within filaments. Experimental models were generated by fitting AF3-predicted FAM118B monomers into the electron density maps and refining them to a good fit.

**Table 1:** CryoEM data collection, refinement and validation statistics

	1. FAM118B full length, two protomers (EMD-53487), (PDB 9R0P)	2. FAM118B full length, five protomers (EMD-53488), (PDB 9R0S)	3. FAM118B residues 24–334, two protomers (EMD-53555), (PDB 9R3E)	4. FAM118B residues 24–334, six protomers (EMD-53556)
**Data collection and processing**				
Magnification	150,000	150,000	105,000	105,000
Voltage (kV)	300	300	300	300
Electron exposure (e− per Å^2^)	43.70	43.70	40.18	40.18
Defocus range (μm)	−1.6 to −2.6	−1.6 to −2.6	−0.8 to −2.8	−0.8 to −2.8
Pixel size (Å)	0.830	0.830	0.839	0.839
Symmetry imposed	None	None	None	None
Initial particle images (no.)	531,673	531,673	2,739,584	2,739,584
Final particle images (no.)	368,283	111,156	289,932	289,932
Map resolution (Å)	4.07	4.96	3.8	8.7
FSC threshold	0.143	0.143	0.143	0.143
Map resolution range (Å)	3.8–6.5	4.8–7.0	3.6–7.3	7.8–10
**Refinement**				
Initial model used (PDB code)	AF3	AF3	AF3	
Model resolution (Å)	4.5	8.0	7.9	
FSC threshold	0.5	0.5	0.5	
Map sharpening *B* factor (Å^2^)	−170	−247	−266	
Model composition				
Non-hydrogen atoms	7,209	12,015	4,792	
Protein residues	897	1,495	596	
Ligands	0	0	0	
*B* factors (Å^2^)				
Protein			465	
Ligand			0	
R.m.s.d.				
Bond lengths (Å)	0.640	0.640	0.003	
Bond angles (°)	1.060	1.040	0.612	
**Validation**				
MolProbity score	1	1	2.53	
Clashscore	2.6	2.5	16.9	
Poor rotamers (%)			5.6	
Ramachandran plot				
Favored (%)	94	94	96.11	
Allowed (%)	5	6	3.89	
Disallowed (%)	1	0	0	

### FAM118B has a noncanonical sirtuin fold

The part of each FAM118B protomer that is well resolved in the density maps is limited precisely to residues 28–325 for both FAM118B and FAM118B_24__–334_, indicating that the flexibility of N and C extremities extends slightly beyond the parts truncated in the shorter construct (Fig. 2g). Noteworthily, the resolved region matches exactly the part of the AF3 model with good predicted local distance difference test (pLDDT) values. The final structures of all individual protomers of both FAM118B and FAM118B_24__–334_ are similar to each other and align well with the AF3 model.

As expected, the FAM118B sirtuin domain comprises the large Rossmann-fold subdomain and a small α-helical subdomain but lacks a zinc-binding module (Fig. 2h). The zinc-binding module, missing from FAM118B and other SIR2_2 proteins, is required for the deacetylation activity of canonical sirtuins^10,44^, stabilizing the overall fold and/or facilitating protein substrate binding. Given that SIR2_2 proteins, including FAM118B, are likely ancestral to canonical sirtuins^39^, the zinc-binding module may be a later development associated with the apparent shift from NADase to protein deacetylation activity and from homomultimerization, which may help stabilize the fold, to a merely monomeric or dimeric state^45,46^.

### FAM118B filaments are formed through a head-to-tail interaction

**Fig. 3:**
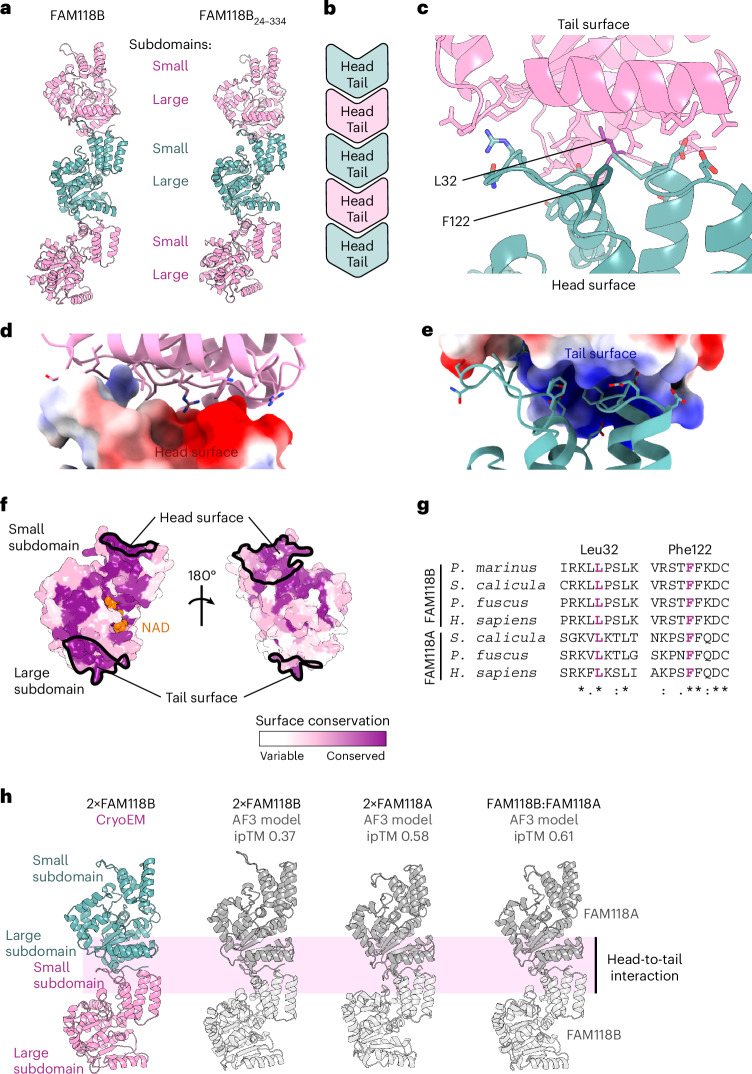
FAM118B forms filaments through conserved head and tail surfaces. **a**, Fragments of filaments of full-length FAM118B and FAM118B_24–334_ composed of three protomers, with alternating protomers colored pink or teal. Three consecutive subunits from cryoEM reconstruction of a five-protomer or six-protomer filament fragment were used, respectively. **b**, Schematic representation of head-to-tail filament assembly. **c**, Zoomed-in view of a cryoEM model of the FAM118B_24__–334_ filament fragment composed of two protomers showing head-to-tail interaction interface between consecutive protomers. Side chains that might participate in the interaction are shown as sticks but their position might be inaccurate because of limited resolution. Central F122 and L32 residues substituted in this study are highlighted in a darker shade. **d**,**e**, Filament interface from **c** with either head (**d**) or tail (**e**) surface colored according to estimated electrostatic potential (red, negative charge; blue, positive charge). **f**, AF3 model of folded part of human FAM118B (residues 28–334) colored according to sequence conservation among FAM118-clade members (same as in Fig. 1c, top). **g**, Fragments of a multiple-sequence alignment of FAM118B and FAM118A from various vertebrates around the central residues of tail and head surfaces. Sequences from the following species were used: jawless fish (*Petromyzon* *marinus*), cartilaginous fish (*S*. *canicula*), amphibian (*P*. *fuscus*) and mammal (*H*. *sapiens*). **h**, Structural comparison of a fragment of full-length FAM118B cryoEM reconstruction showing a head-to-tail arrangement with AF3 models of indicated homodimers or heterodimers. The ipTM score for each AF3 model is provided. AF2 models of the same complexes are provided in Extended Data Fig. 8. Details of AF3 and AF2 models are provided in Extended Data Fig. 9.

As mentioned above, individual FAM118B protomers are arranged into filaments in our cryoEM maps (Fig. 2a–f). Both FAM118B and FAM118B_24__–334_ structures show essentially the same assembly where each successive protomer is translated forward and rotated by approximately 40° around the longitudinal axis, creating a helical arrangement (Fig. 3a). Longer filaments seen in EM images (Fig. 2a,d) likely involve further iterations of the same interaction mode.

To assemble into FAM118B filaments, a part of the small α-helical subdomain (the head surface) of one protomer binds to the opposite end of the large subdomain (the tail surface) on a neighboring protomer (Fig. 3a,b).

The large head-to-tail interaction interface (>1,000 Å^2^) is predominantly polar in nature, featuring numerous potential hydrogen bonds and salt bridges, along with several hydrophobic residues (Fig. 3c). It likely has an electrostatic component, with a negatively charged head (Fig. 3d) and a positively charged tail (Fig. 3e) surface.

### Filament formation is a conserved feature of FAM118s

The head and tail surfaces account for a substantial part of the surface conserved among FAM118-clade proteins (Fig. 3f). Moreover, at the sequence level, key head and tail residues, notably including the central hydrophobic residues F122 and L32, are well conserved among FAM118B and FAM118A proteins across vertebrates (Fig. 3g), suggesting that the ability to form filaments with this particular topology is their shared, ancestral feature. Conserved patches extend beyond the head and tail surfaces (Fig. 3f), indicating additional interactions conserved among FAM118s.

**Extended Data Fig. 8:**
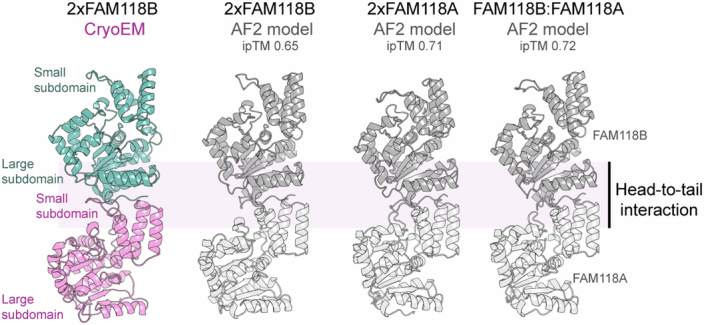
Structural comparison of head-to-tail dimers from cryoEM structure and AlphaFold2 modeling. A head-to-tail dimer derived from the cryoEM structure of full-length human FAM118B compared to AlphaFold2 (ColabFold v1.5.5) models of FAM118B or FAM118A homodimers or a FAM118B:FAM118A heterodimer. The image of the cryoEM dimer is the same as in Fig. 3h. Interface predicted template modeling (ipTM) score values for the best out of five calculated models are provided. Model details are provided in Extended Data Fig. 9.

**Extended Data Fig. 9:**
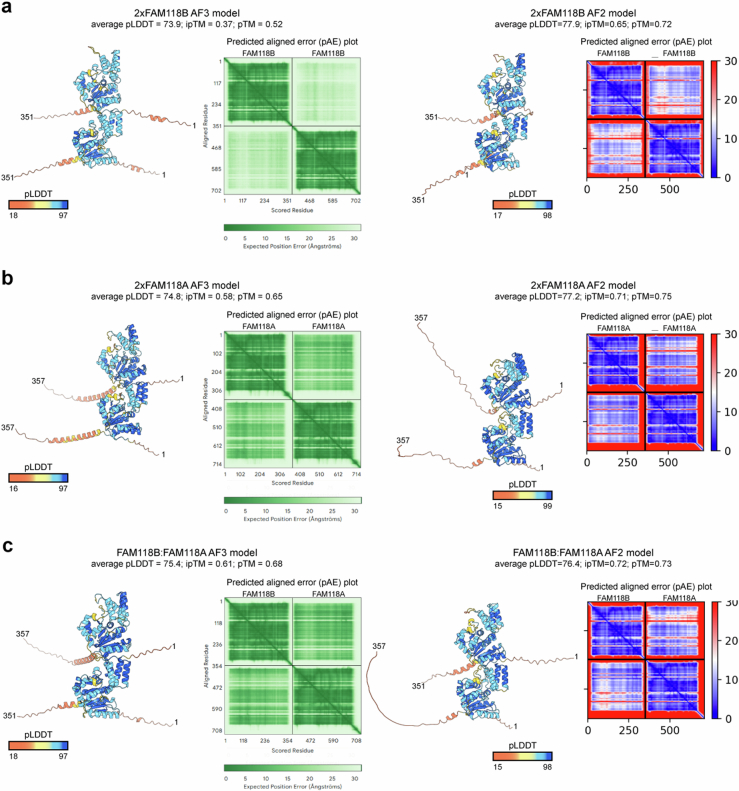
AlphaFold modeling of FAM118B and FAM118A homodimers and FAM118B:FAM118A heterodimer. AlphaFold 3 (AlphaFold server) or AlphaFold 2 (ColabFold v1.5.5) models are presented on left and right, respectively. The top out of five calculated models is presented in each case. The models are presented like in Extended Data Fig. 1, coloured according to per-residue predicted local distance difference test (pLDDT) score. First and last residues in a chain are numbered. The average pLDDT score, interface predicted template modeling (ipTM) score, and predicted template modeling (pTM) score are also provided, together with a predicted aligned error (pAE) plot, coloured according to the indicated scale. **a**, Models of human FAM118B homodimer. **b**, Models of human FAM118A homodimer. **c**, Models of human FAM118B:FAM118A heterodimer. These models are used in Fig. 3h and Extended Data Fig. 8.

We also tested whether AF2 or AF3 could predict the filamentous arrangement to assess whether it is encoded in the evolutionary and structural features that these algorithms recognize. Importantly, our cryoEM structures were not included in the PDB training sets for either AF version and, as mentioned, no previously characterized sirtuin forms filaments with this topology. Modeling human FAM118A or FAM118B homodimers or an FAM118A:FAM118B heterodimer, we found that AF2 and AF3 reproducibly predict a head-to-tail dimer that can be extrapolated to a filament (Fig. 3h for AF3 and Extended Data Fig. 8 for AF2). The interface predicted template modeling (ipTM) scores and other confidence metrics of these predictions—falling within an intermediate range (Extended Data Fig. 9)—would have left these predictions uncertain without experimental validation; in our case, these models align with and support the experimental data. Of note, a recent large-scale AF2-based analysis of homomultimerization within the human proteome predicted human FAM118A (but not FAM118B) as potentially multimerizing with a translational symmetry^47^, consistent with our predictions.

### Filament formation can be prevented by mutating head or tail surfaces

As cryoEM showed that both FAM118B and FAM118B_24__–334_ have similarly strong filamentation tendencies—suggesting that the N-terminal and C-terminal tails are dispensable for this property—we used the FAM118B_24__–334_ construct to investigate filament formation in solution using biophysical techniques.

**Fig. 4:**
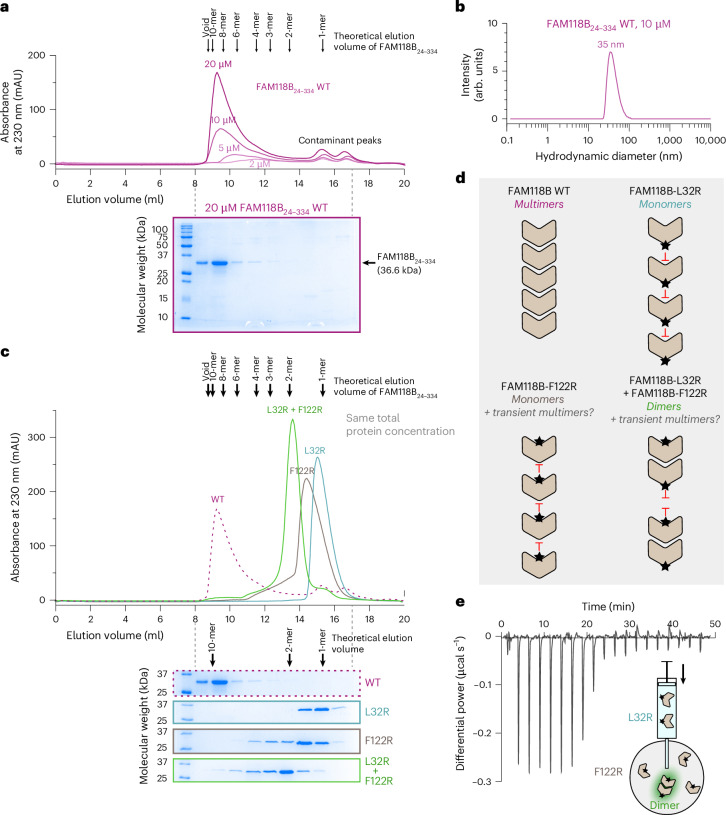
FAM118B forms head-to-tail multimers in vitro*.* **a**, Analytical SEC of recombinant FAM118B lacking N and C extremities (FAM118B_24__–334_) injected at the indicated concentrations. The chromatogram shows superposed traces from independent runs performed with varying injection concentrations. SDS–PAGE analysis of fractions from the run of the sample injected at 20 µM is provided underneath. Small late peaks, labeled as ‘contaminant peaks’, correspond to fractions without detectable FAM11B on SDS–PAGE. Arrows above the chromatogram indicate coarse estimates of elution volumes of different multimeric states calculated on the basis of a calibration curve generated with globular protein standards. **b**, DLS analysis of FAM118_24__–334_ at 10 µM. **c**, Analytical SEC of indicated mutant forms of FAM118_24__–334_ at 20 µM. In the case of a mixture of L32R and F122R mutants (labeled L32R + F122R), each mutant was at 10 µM, resulting in a total concentration of 20 µM. The chromatogram shows superposed traces from independent runs of indicated samples, each accompanied by SDS–PAGE. The result for WT FAM118_24__–334_ was reused from **a**. Arrows are coarse estimates of expected elution volumes like in **a**. **d**, Schematic illustrating conclusions from SEC analysis. Mixing a head-surface (F122R) and a tail-surface (L32R) mutant reconstitutes a head-to-tail dimer, explaining the shift of SEC peak for the mutant mixture relative to either mutant alone. **e**, ITC isotherm of FAM118B_24__–334_-L32R binding to FAM118B_24__–334_-F122R. Inset: schematic representation of experiment. An integrated enthalpy change plot and estimated binding parameters are provided in Extended Data Fig. 10. Experiments were independently repeated *n* = 3 times using the same protein samples, producing similar results. Source data

We first analyzed the wild-type (WT) variant of FAM118B_24__–334_ in analytical size-exclusion chromatography (SEC) runs at increasing injection concentrations, observing progressively earlier elution and ultimately approaching the void volume at the injection concentration of 20 µM (Fig. 4a). The observed elution profiles indicate a large apparent molecular weight (MW), likely corresponding to an average of >8 protomers (based on a coarse estimate using globular protein standards) for the injection concentration of 20 µM. These observations are consistent with concentration-dependent filament formation^42^ and, given that SEC dilutes the injected sample approximately tenfold, appreciable filaments form already at submicromolar to low-micromolar effective concentrations. We could not further investigate this phenomenon using SEC coupled to multiangle light scattering (SEC–MALS) because the protein adhered to high-performance liquid chromatography-type columns used by this method.

Analyzing instead WT FAM118B_24__–334_ with dynamic light scattering (DLS), we observed, at 10 µM, a well-defined main peak corresponding to the hydrodynamic diameter of around 35 nm (Fig. 4b). Given that a single FAM118B_24__–334_ protomer is about 6 nm long and 5 nm wide, this suggests oligomers of <10 subunits on average at the analyzed concentration.

Next, to disrupt filament formation, we designed two substitutions, F122R and L32R, inserting bulky residues into the head or tail interface (Fig. 3c). Analytical SEC at the injection concentration of 20 µM showed that each mutant in isolation is largely monomeric; L32R eluted as a narrow monomeric peak, while F122R showed slight tailing, suggesting residual filament formation. Mixing the two mutants at a total injection concentration of 20 µM produced a sharp peak at the expected dimer volume, consistent with a single head-to-tail interaction (Fig. 4d).

**Extended Data Fig. 10:**
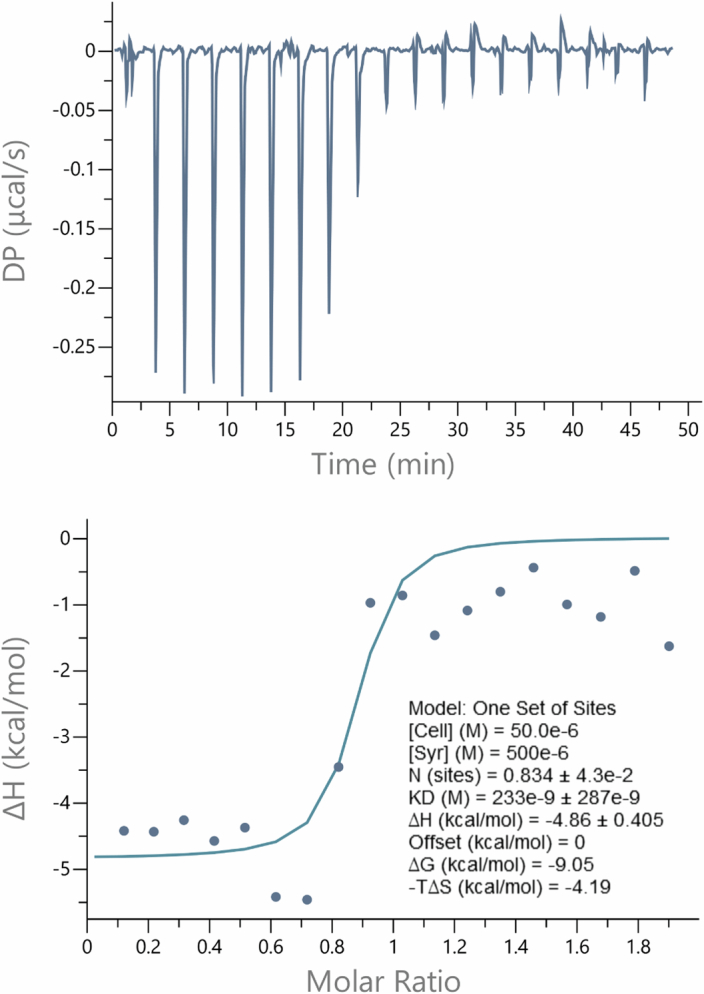
Isothermal titration calorimetry (ITC) analysis of binding between tail and head mutants of FAM118B. ITC binding isotherm (*top*) and integrated enthalpy change curve (*bottom*) for an experiment (also shown in Fig. 4e) in which FAM118B_24-334_ L32R was injected at 500 µM into FAM118B_24-334_ F122R at 50 µM. In the bottom curve, the signal is corrected by subtracting heat of dilution (buffer into buffer, L32R into buffer, and buffer into F122R). Binding parameters estimated assuming a one-binding-site model are provided with uncertainty ranges corresponding to the uncertainty of fitting. The *K*_D_ value – which appears to be in a low-to-high nanomolar range – cannot be precisely determined due to the high steepness of the curve (high *c* value) characteristic of high-affinity interactions. A representative experiment of *n* = 3 similar repeats performed with the same protein samples is shown.

We further analyzed the interaction between the two mutants, which mimics a single link in a WT FAM118B chain, using isothermal titration calorimetry (ITC). The sharply decaying exothermic peaks observed when injecting L32R into F122R are consistent with an approximately 1:1 stoichiometry and high-affinity binding (Fig. 4e and Extended Data Fig. 10), with a dissociation constant (*K*_D_) likely in a low-nanomolar to high-nanomolar range, although the exact value could not be determined because of high affinity.

Overall, these results confirm that FAM118B possesses two independent surfaces capable of interacting with each other with high affinity. In the WT protein, these interactions mediate the self-assembly of individual protomers into multimers in a submicromolar or low-micromolar concentration regime.

### FAM118B forms multimers in living human cells

**Fig. 5:**
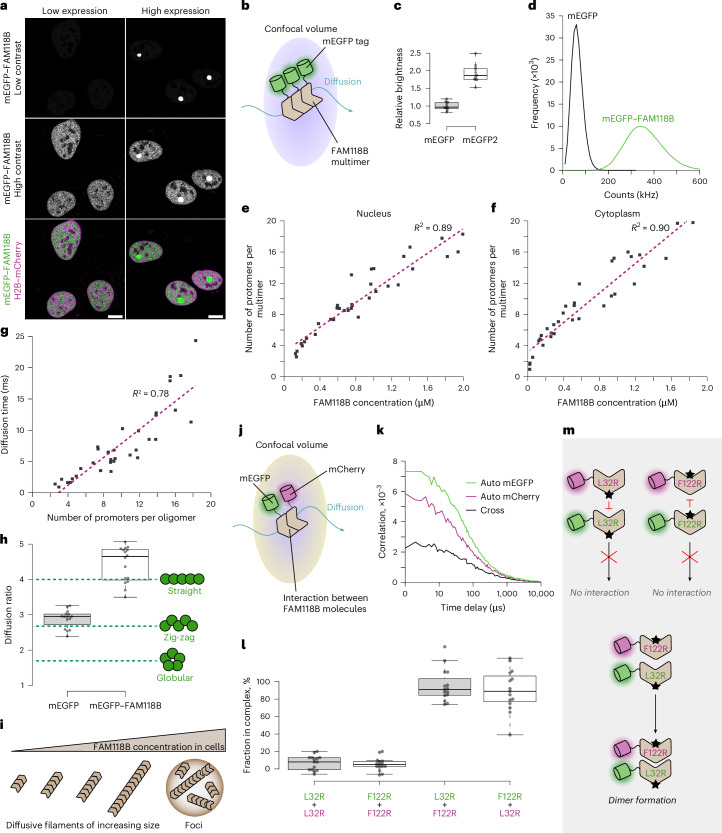
FAM118B forms head-to-tail multimers in living human U2OS cells. **a**, Representative confocal images of cells transiently transfected with mEGFP–FAM118B WT, showing low-to-medium expression (left) with a diffuse distribution or high expression (right) with additional nuclear focus formation. mCherry-tagged histone H2B was used as a nuclear marker. A representative image from *n* = 15 images of cells with a similar pattern is shown. Scale bars, 5 μm. **b**, Schematic illustrating the principle of PCH analysis, which monitors homomultimerization by assessing the brightness of fluorescently tagged protein complexes diffusing through confocal volume. **c**, Positive control showing that PCH analysis gives brightness for an mEGFP tandem (mEGFP2) that is approximately twice that of a single mEGFP (*n* = 12 cells per condition). **d**, Sample PCHs for free mEGFP and mEGFP–FAM118B. Fitting these histograms gives access to the number of labeled multimers within confocal volume and their average brightness. **e**, Estimation of multimer size as a function of FAM118B concentration in the nucleus of cells with diffusive FAM118B distribution. Each point corresponds to a measurement in a different cell from *n* = 35 cells. Oligomeric state was estimated by comparing brightness of fluorescently tagged FAM118B oligomers to that of free mEGFP. **f**, Same as in **e** but performed in the cytoplasm with *n* = 34 cells. **g**, Diffusion time of multimers in nucleus as a function of multimer size for *n* = 35 cells. Diffusion time was estimated from the autocorrelation of raw fluorescence fluctuation data used for PCH. In **e**,**f**, and **g**, the dotted purple line is a linear least square regression of experimental data with associated *R*^2^ value. **h**, Diffusion ratios from fluorescence correlation spectroscopy, defined as the diffusion coefficient of a smaller multimer divided by that of a larger multimer, were compared between mEGFP–FAM118B (2-mer/10-mer) and a flexible fusion of multiple mEGFPs (1-mer/5-mer) and evaluated against theoretical values from Perrin’s equations for different shapes (green dotted lines). The box plot shows the minimum (lower whisker), first quartile (lower box edge), median (thick line), third quartile (upper box edge) and maximum (upper whisker). The data points are from *n* = 15 cells for the multiple-mEGFP control and *n* = 16 cells for mEGFP–FAM118B. **i**, Schematic illustrating apparent multimerization of mEGFP–FAM118B in cells as a function of concentration. Increasing the concentration increases the average filament length, with filaments remaining diffusive up to a threshold above which most protein tends to segregate into distinct foci. **j**, Schematic illustrating principle of FCCS, which assesses the interaction between two proteins tagged with different fluorophores by monitoring their codisplacement within confocal volume. **k**, Sample autocorrelation and cross-correlation curves in a cell coexpressing mEGFP–FAM118B-L32R and mCherry–FAM118B-F122R. **l**, Proportion of the less concentrated construct in complex with the other construct estimated from the amplitudes of cross-correlation curves relative to those of autocorrelation curves. Label colors indicate fluorescent tags: green for mEGFP and red for mCherry (*n* = 15 cells per condition). **m**, A possible interpretation of FCCS experiment involving head-to-tail dimer formation when coexpressing a head-surface (F122R) and tail-surface (L32R) mutant of FAM118B but not two differently tagged copies of the same mutant. All box plots show the individual measurements (points, each corresponding to a different cell), minimum (lower whisker), first quartile (lower box edge), median (thick line), third quartile (upper box edge) and maximum (upper whisker). Experiments in **a**,**c**–**h**,**k**,**l** were repeated independently *n* = 3 times with similar results. Source data

To study FAM118B multimerization in living cells, we expressed full-length FAM118B fused at its N terminus to monomeric enhanced green fluorescent protein (mEGFP) in human U2OS cells. We capitalized on the range of expression levels achieved in transiently transfected cells to study this protein at varying concentrations (Fig. 5a).

Above a certain level of expression, the protein partly localizes to distinct nuclear foci (Fig. 5a, right), which we address later in this article. Below this threshold, it is diffusively distributed, primarily in the nucleus, with a minor cytoplasmic population (Fig. 5a, left). It is within the lower-expressing cells, which likely better reflect physiological conditions, that we probed the size and shape of FAM118B complexes using various approaches.

First, we monitored their multimerization using photon-counting histogram (PCH) analysis, which fits photon count distributions of mEGFP-tagged complexes diffusing in and out of the confocal volume to extract brightness and concentration (Fig. 5b). Assuming that the brightness of a complex scales linearly with the number of mEGFP tags, PCH allows the estimation of the number of protomers within a multimeric species independently of diffusion time. We verified that a tandem fusion of two mEGFPs is approximately twice as bright as a single mEGFP (Fig. 5c). Moving on to mEGFP–FAM118B analysis (Fig. 5d), we performed PCH measurements separately in the nucleus and the cytoplasm, revealing that, in both compartments, FAM118B forms homomultimers composed of 2–20 tagged protomers, scaling linearly with protomer concentration ranging from approximately 30 nM to 2 µM (Fig. 5e,f).

The comparable PCH results obtained for the nucleus and the cytoplasm suggest that FAM118B multimerization is not affected by association with the previously reported^48^ nuclear interactors such as coilin (known to be well expressed in U2OS cells^49^) or any other factors unequally distributed between the nucleus and the cytoplasm and present at sufficient levels in these cells. In fact, PCH shows a broadly similar increase in multimer size across the submicromolar to low-micromolar range to that observed for purified recombinant protein by SEC in vitro (Fig. 4a), suggesting that FAM118B multimerization in cells reflects an intrinsic property of this protein.

### FAM118B multimers detected in cells are consistent with filaments observed in vitro

We next wanted to verify that the FAM118B multimers detected in cells correspond to filaments similar to those characterized in vitro. Another possibility is the aggregation of misfolded protein, although the small, well-defined size of the diffuse multimers argues against this. To further distinguish between these possibilities, we conducted measurements that provide information on particle shape. Whereas filaments are rod-like, aggregates are expected to approximate a globule, except in a specific case of amyloid formation^50^. By autocorrelating the raw fluorescence fluctuations acquired for PCH analysis in the nucleus, we were able to quantify the average time spent by the multimers within the confocal volume and plot it as a function of estimated multimer size. These measurements revealed a linear correlation between the diffusion time and the protomer number (Fig. 5g), which fits better a rod-like filament—for which the hydrodynamic drag is expected to be proportional to length—than a globular aggregate^51^.

As a second approach, we used fluorescence correlation to calculate the ratio of diffusion speeds of mEGFP–FAM118B multimers containing two versus ten protomers. As a control, we calculated the corresponding ratio for a single mEGFP versus five mEGFP molecules linearly fused through flexible linkers. The comparison of these diffusive ratios to the theoretical predictions of the Perrin’s equation indicates that neither the mEGFP fusions, as shown previously^51,52^, nor the FAM118 multimers adopt a globular shape (Fig. 5h). Instead, both show an elongated conformation with FAM118B being straighter than the flexible mEGFP pentamer. These findings further support that cellular FAM118B forms filament-like structures rather than aggregates.

Overall, the cellular data support that FAM118B forms extended filaments that grow in protomer number with concentration and ultimately condense into foci at high overexpression (Fig. 5i).

### FAM118B protomers form head-to-tail interactions in cells

Continuing with the live-cell analysis, we wanted to verify that the head-to-tail association, which is at the basis of filament formation by the WT FAM118B protein in vitro, is formed within living cells. To do so, we assessed the interaction between FAM118B L32R or F122R mutants differentially tagged with either mEGFP or mCherry using fluorescence cross-correlation spectroscopy (FCCS), which detects protein–protein association through their correlated displacement within the confocal volume (Fig. 5j,k). These mutants were exclusively diffusively distributed in cells, localizing mainly to the nuclei. When coexpressing the same mutant (either L32R or F122R) with two different tags, we did not observe any interaction, consistent with abolished multimerization (Fig. 5l). In contrast, the mEGFP–FAM118B-L32R:mCherry–FAM118B-F112R pair or the mEGFP–FAM118B-F122R:mCherry–FAM118B-F112R pair associated strongly in cells. These data align with the head-to-tail interaction configuration observed in vitro (Fig. 5m).

### FAM118 foci formed at high expression require filament assembly and are distinct from known subnuclear domains

**Fig. 6:**
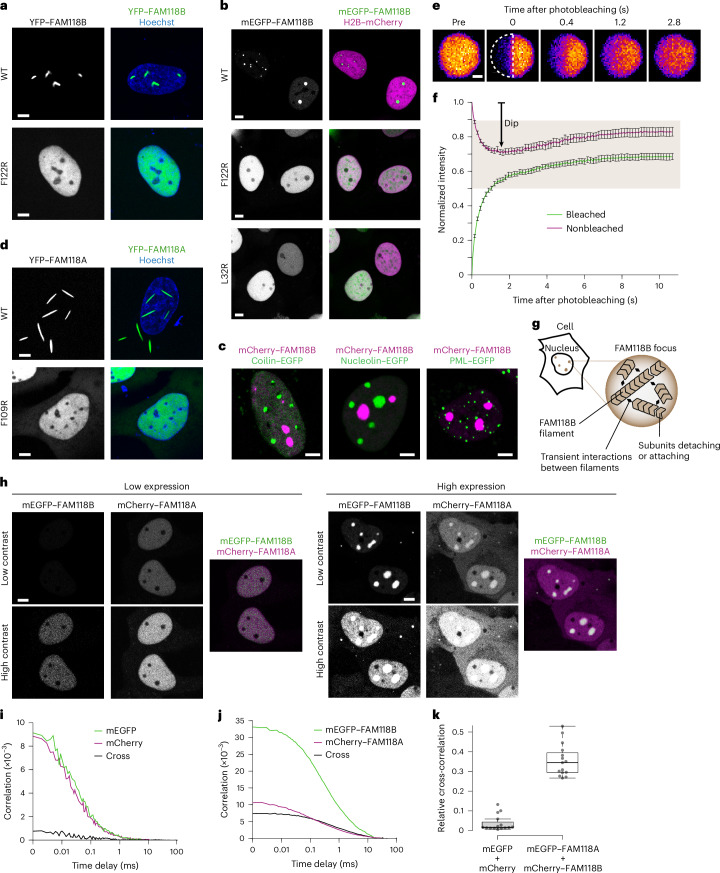
FAM118B and FAM118A form foci at high expression levels and interact together in living U2OS cells. **a**, Representative confocal images of nuclei of cells overexpressing YFP-tagged FAM118B in WT or F122R forms. In the merged image, the nucleus is counterstained with Hoechst. **b**, Same as in **a** but for YFP–FAM118A WT or F109R. **c**, Same as in **a** but for mEGFP-tagged FAM118B WT, F122R or L32R, with nuclear localization indicated with mCherry-tagged histone H2B instead of Hoechst. **d**, Representative confocal images of nuclei of cells coexpressing mCherry–FAM118B with various EGFP-tagged markers of nuclear domains: coilin (Cajal bodies), nucleolin (nucleolus) or PML isoform 4 (PML bodies). **e**, Representative images of FRAP of half of an mCherry–FAM118B nuclear focus. Dashed, half circle indicates the bleached area. Scale bar, 1 µm. **f**, Half-focus FRAP curves obtained by quantifying fluorescence in bleached or nonbleached parts from images like those in **d**. Curves are the mean ± s.d. of 16 foci from different cells. The beige plot area represents the range of dip depths in the nonbleached half reflecting preferential diffusion within versus beyond a focus. **g**, Schematic illustrating possible mechanism of focus formation by FAM118B, where individual protomers reversibly assemble into filaments, which further associate, either directly or through another biomolecule, to segregate in a droplet-like focus at very high expression levels in cells. **h**, Representative confocal images of nuclei of cells transiently coexpressing mEGFP–FAM118B and mCherry–FAM118A, with low-to-medium expression (left) with exclusively diffusive distribution or high expression (right) with additional focus formation, predominantly in the nucleus. **i**, Autocorrelation and cross-correlation FCCS curves in a negative control cell coexpressing free mEGFP and mCherry fluorophores. **j**, Same as in **i** but in a cell coexpressing mEGFP–FAM118B and mCherry–FAM118A. **k**, Cross-correlation amplitude relative to mean autocorrelation curve amplitude. The strong cross-correlation signal in cells coexpressing mEGFP–FAM118A and mCherry–FAM118B compared to negative control demonstrates the interaction between FAM118A and FAM1118B in living cells (*n* = 15 cells per condition). The box plot shows the minimum (lower whisker), first quartile (lower box edge), median (thick line), third quartile (upper box edge) and maximum (upper whisker). For images of cells in **a**–**d**,**h**, the provided images are representative of *n* = 15 images of cells with a similar pattern. In all cellular images except for the foci in **e**, the scale bar corresponds to 5 μm. Experiments in **a**–**f**,**h**–**k** were repeated independently *n* = 3 times with similar results. Source data

As already discussed, WT mEGFP–FAM118B can partly separate into foci above a certain overexpression level (Fig. 5a,i). Although the required protein concentrations are likely nonphysiological, we investigated this process as an additional window into the intrinsic self-association potential of these proteins. In fact, irrespective of the fluorescent tag used (mEGFP, yellow fluorescent protein (YFP) or mCherry), both FAM118B and FAM118A form foci above a certain concentration threshold, suggesting that the process is not triggered by fluorescence tagging, although it does seem to be modulated by it (Fig. 6a–c). Paralog identity also affects focus morphology, as YFP–FAM118B and YFP–FAM118A foci differ sharply in shape despite having the same tag (Fig. 6a,d, top). Most FAM118B and FAM118A foci are nuclear, although occasional cytoplasmic ones are also observed.

To test whether focus formation requires filament formation, we examined the subcellular localization of filament-deficient FAM118 mutants. Introducing the F122R substitution, which impairs filament formation in vitro (Fig. 4c), into YFP–FAM118B (Fig. 6a, bottom), or the equivalent F109R substitution into YFP–FAM118A (Fig. 6d, bottom) led to an exclusively diffusive distribution across all examined expression levels. These findings were further confirmed using mEGFP-tagged FAM118B, where foci were completely lost in the tail (L32R) or head (F122R) mutants (Fig. 6b, middle and bottom).

To determine whether the observed foci colocalize with known subnuclear domains, including Cajal bodies (reported to require FAM118B for assembly^48^), nucleoli and PML bodies, we coexpressed mCherry–FAM118B WT with EGFP-tagged versions of their respective marker proteins: coilin, nucleolin and PML isoform 4 (Fig. 6c). Although all three markers formed distinct nuclear foci as expected, none overlapped with mCherry–FAM118B foci, indicating that these correspond to distinct subnuclear domains.

### FAM118B foci are demixed from their environment and show high internal dynamics

Lastly, to probe the dynamics of FAM118B within foci and the extent to which the foci remain separated from their environment, we used a recently described ‘half-focus’ fluorescence recovery after photobleaching (FRAP) assay (Fig. 6d,e)^53^. mEGFP–FAM118B was highly mobile within the foci, arguing against misfolded aggregates, expected to show little dynamics^54^. Furthermore, monitoring the fluorescence reequilibration between the bleached and unbleached area showed that, in contrast to their high internal fluidity, the FAM118B foci display little exchange with the rest of the nucleoplasm. More specifically, the mean dip observed for the unbleached area on the fluorescence redistribution curve was equal to 0.29 (Fig. 6e, arrow), significantly above the value expected if proteins freely diffused in and out the foci (*P* = 10^−9^), indicating a diffusion barrier at the foci border.

Together, these results suggest that the FAM118B and FAM118A foci observed at high overexpression are dynamic higher-order assemblies composed of filaments and separated from their environment (Fig. 6g). Given their micrometer-scale dimensions, each focus likely contains numerous filaments.

### FAM118B and FAM118A interact in cells

Next, we investigated whether human FAM118A and FAM118B physically interact in cells by transiently coexpressing mEGFP-tagged FAM118B and mCherry-tagged FAM118A in human U2OS cells. As for FAM118B alone, expression levels varied; cells with lower expression showed exclusively diffusive distribution of both proteins, while highly expressing cells displayed large nuclear foci alongside a diffusive subpopulation (Fig. 6h).

The two paralogs colocalized in foci at high expression but we sought to investigate whether the two proteins also show signs of interaction in focus-deficient, lower-expressing cells more closely resembling physiological conditions. Compared to coexpressed free mCherry and mEGFP used as a negative control (Fig. 6i), the two tagged FAM118s showed strong correlated movement in these cells in FCCS (Fig. 6j,k), confirming physical association.

### FAM118B has a potential site for NAD binding and processing

**Fig. 7:**
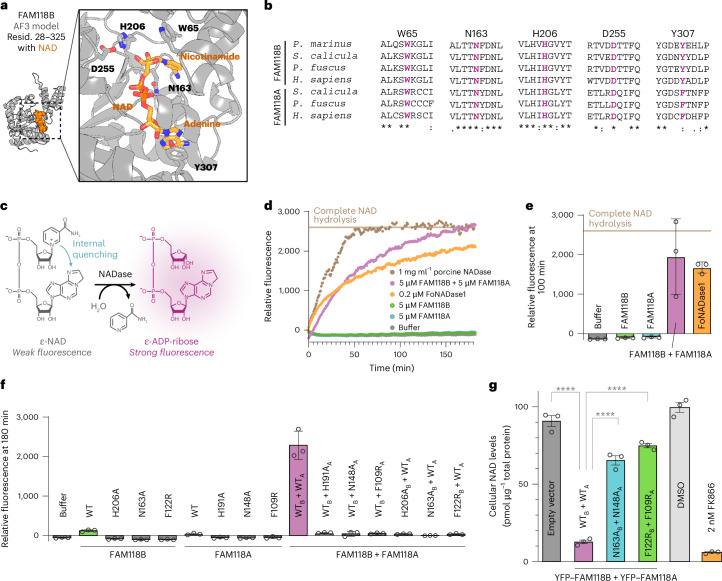
FAM118B:FAM118A interaction leads to synergistic NAD-processing activity. **a**, An AF3 model of human FAM118B (residues 28–325 are shown) bound to NAD (orange) and a zoomed-in view of predicted NAD-binding site with selected side chains shown as sticks. Model details are given in Extended Data Fig. 1c. Another view of model and alignment with cryoEM structures is provided in Supplementary Fig. 1. **b**, Fragments of a multiple-sequence alignment of FAM118B and FAM118A from various species (legend in Fig. 3g) around indicated residues. **c**, Principle behind ε-NAD-based colorimetric NAD-processing assay: fluorescence quenching is relieved when nicotinamide is eliminated by an NADase or related activity. **d**, Relative fluorescence indicating ε-NAD processing monitored over time in a single experimental repeat. A representative result of *n* = 2 independent repeats is shown. The relative fluorescence level approximately corresponding to complete ε-NAD hydrolysis (beige line) was estimated on the basis of the plateau region of porcine NADase curve (100 to 180 min). **e**, Relative fluorescence indicating ε-NAD processing measured at 100 min in *n* = 3 technical repeats involving individually prepared reactions using the same protein stocks. The series was independently repeated *n* = 2 times with similar results. The beige line is the same as in **d**. **f**, Same as in **e** but at 180 min, based on *n* = 3 independent technical replicates using the same protein stocks. For experiments where FAM118B and FAM118A were mixed, the WT or mutant state of either protein is indicated with a ‘B’ or ‘A’ in subscript. FAM118B or FAM118A proteins were present at 5 µM each (total 10 µM for FAM118B + FAM118A). The series was independently repeated *n* = 2 times with similar results. **g**, Normalized levels of NAD in human 293T cells, normalized to total protein amount, determined using colorimetric assay after a 2-h incubation based on *n* = 3 technical replicates using the same cell lysates. A representative set of results from *n* = 4 independent biological replicates of the entire series is shown. Significance was determined using a one-way ANOVA and Tukey’s post hoc test for multiple comparisons. *P* = 7.7 × 10^−8^ for empty vector versus WT_B_ + WT_A_, WT_B_ + WT_A_ versus catalytic mutants and WT_B_ + WT_A_ versus filamentation mutants; *P* = 0.0004 for empty vector versus catalytic mutants; *P* = 0.0089 for empty vector versus filamentation mutants. An unpaired one-tailed *t*-test was calculated for the DMSO control versus FK866 inhibitor-treated cells. *P* = 8.0 × 10^−9^. In the bar graphs in **e**–**g**, individual measurements (circles) and means (top bar edge) are shown, with error bars indicating the s.d. (**e**,**f**) or s.e.m. (**g**). *P ≤ 0.05, **P ≤ 0.01, ***P ≤ 0.001 and ****P ≤ 0.0001. Source data

We moved on to investigating the catalytic activity of FAM118s. To define the potential NAD-binding site, we modeled FAM118B in complex with NAD using AF3, which positioned the substrate in the expected pocket between the large and small subdomains (Fig. 7a, Extended Data Fig. 1c and Supplementary Fig. 1).

Potential NAD-binding and/or catalytic residues include a histidine, H206, and an adjacent aspartate, D255, both from the large subunit (Fig. 7a). A histidine in a similar position is found in many sirtuins and is important for SIRT2 and ThsA ativity^11,27,55^. In our model, H206 is positioned near the 2′ and 3′ hydroxyl groups of the nicotinamide-proximal ribose ring of NAD and might not only interact with but also polarize or deprotonate these groups, which in turn could stabilize the transition state of NAD decomposition where a partial positive charge develops on the ribose as the nicotinamide departs^56^. While the p*K*_a_ of histidine would normally be too high to allow efficient hydroxyl deprotonation, this might change in an appropriate microenvironment. Compared to H206, D255 appears more specific to FAM118s, as it is absent from SIRT2 or ThsA; on the basis of its position within our model, it may directly interact with the ribose hydroxyls of NAD and/or affect these groups indirectly by polarizing H206 to lower its p*K*_a_. Notably, both H206 and D255 are strictly conserved in FAM118B and FAM118A across vertebrates, in line with their potential functional role (Fig. 7b).

Another active-site residue, N163, apparently has a central role in an interaction network that holds together C-terminal and N-terminal regions constituting the NAD pocket (Fig. 7a). Additionally, N163 may stabilize the build-up of the partial positive charge on the ribose during nicotinamide cleavage, as shown for the equivalent residue in yeast Sir2 (refs. ^23,57^). N163 is highly conserved in FAM118B and FAM118A (Fig. 7b), as well as other sirtuins, including SIRT2 and ThsA.

The NAD-bound FAM118B model suggests that two aromatic residues, W65 (small subdomain) and Y307 (large subdomain), may interact with the nicotinamide and adenine rings of NAD, respectively (Fig. 7a). W65, which occupies a similar position to F273 in SIRT1, may contribute to both NAD binding and its cleavage, as nicotinamide desolvation promoted by a hydrophobic environment facilitates the latter process^56,58^. The similarly positioned phenylalanine of canonical sirtuins seemingly switches from interacting with nicotinamide in the NAD-bound state to itself occupying the nicotinamide site in the absence of NAD^59^ and our NAD-free FAM118B cryoEM density, albeit at moderate resolution, is consistent with W65 sliding into the predicted nicotinamide pocket in the absence of NAD (Extended Data Fig. 5 and Supplementary Fig. 1), which could enhance product release or serve another role. Again, W65 and Y307 are conserved in FAM118B and FAM118A, although Y307 is sometimes replaced by a similar phenylalanine (Fig. 7b).

### Functional partnership between FAM118B and FAM118A unleashes NAD-processing activity in vitro and in cells

To experimentally test the ability of FAM118s to process NAD, we used a fluorometric assay based on an NAD analog, ε-NAD, whose fluorescence increases when the nicotinamide moiety is released through hydrolysis or similar process^60,61^ (Fig. 7c). The two extra atoms on the adenosine base of ε-NAD should be accommodated within FAM118s without steric clashes (Supplementary Fig. 2).

Using this approach, we found that 5 µM purified His_6_-tagged full-length FAM118B or FAM118A showed no or very low NAD-processing activity individually (Fig. 7d,e). However, mixing the two paralogs, each at 5 µM, produced a robustly detectable NAD-processing activity that was dramatically increased compared to that of individual proteins. Nonetheless, this elevated activity remained relatively weak compared to a previously characterized extracellular NADase from the fungus *Fusarium* *oxysporum* f.sp. *cubense* race 1 (FoNADase1)^62^, which achieved a similar effect at a much lower concentration of 0.2 µM FoNADase1. We also compared these activities to a standard positive control, commercial porcine brain NADase^63,64^, a large amount (1 mg ml^−1^) of which was used to define complete NAD hydrolysis.

The elevated NAD-processing activity observed upon mixing FAM118B and FAM118A was prevented if either FAM118A or FAM118B was mutated, targeting the mentioned histidine or asparagine residues located in their active sites (H206A or N163A in FAM118B or H191A or N148A in FAM118A) (Fig. 7f). Intriguingly, the activity was also abolished when the ability of either protein to form head-to-tail interactions was disrupted through the F122R substitution in FAM118B or the equivalent F109R substitution in FAM118A. These results suggest that, to unleash NAD processing, not only must both paralogs be present, but each must also be able to at least bind and potentially process NAD and form head-to-tail interactions. This in turn may suggest that the active form of these enzymes corresponds to a mixed FAM118B:FAM118A filament or a higher-order structure composed of homotypic FAM118B-only and FAM118A-only filaments.

Lastly, to validate the observed activity, we tested whether the FAM118B:FAM118A combination is active in depleting NAD in human 293T cells. Indeed, simultaneously overexpressing both YFP-tagged proteins in their WT versions led to severely decreased cellular NAD levels (approximately 15% of empty-vector control) as measured using a cycling colorimetric NAD/NADH assay (Fig. 7g). In contrast, co-overexpressing catalytically inactive (FAM118B-N163A and FAM118A-N148A) or filamentation-deficient (FAM118B-F122R and FAM118A-F109R) variants of both proteins resulted in only a modest NAD decrease to 70–80% of the control levels (Fig. 7g), despite a similar expression of WT and mutant YFP–FAM118 variants (Supplementary Fig. 3). These cellular data demonstrate that FAM118B and/or FAM118A are active in processing NAD in human cells, in a manner dependent on their active site(s) and the ability to form head-to-tail interactions.

## Discussion

While canonical mammalian SIRT sirtuins and, more recently, bacterial sirtuins have been extensively studied, FAM118A and FAM118B—the only human sirtuins beyond SIRTs—have garnered little attention. To date, FAM118B has been the subject of only two studies. One implicated it in Cajal body formation, the production of small nucleolar ribonucleoproteins and overall cell viability^48^. Another, published in its final form while our study was in revision, proposed renaming FAM118B to ‘SIRal’ (for SIR antiphage-like) to highlight its similarity to bacterial sirtuins and its involvement in what is emerging as the ancestral sirtuin function: immunity against pathogens^39^. In the same article, mammalian FAM118B was shown to possess weak NAD-processing activity (detectable only in the presence of chemical crowding agent PEG) and be required—together with this activity—for toll-like receptor-dependent innate immune response in mouse and human cells. The authors speculated that the apparent PEG-dependent increase in FAM118B activity might indicate oligomerization-dependent activation, a prescient hypothesis that remained unexplored.

Here, we argue that FAM118s, while being indeed more closely related to bacterial antiphage sirtuins than to human SIRTs (Fig. 1a,b and Extended Data Fig. 2), belong to a separate clade of vertebrate-specific proteins distinguished by distinct patches of surface residues absent from nonvertebrate SIRal proteins and other sirtuins (Fig. 1c). We further show that, remarkably, FAM118B uses parts of this conserved surface to spontaneously assemble into helical filaments, which we resolved using cryoEM in vitro and detected with fluorescence-based techniques in living cells (Figs. 2–5). Bacterial sirtuins ThsA and Avs5 also assemble into filaments but do so in topologically distinct ways (helical stacking of homotetrameric or homodimeric building blocks in ThsA and Avs5, respectively^32,38^ versus head-to-tail alignment of monomers in FAM118B), indicating independent evolution of a similar property in these different sirtuin lineages.

Interestingly, in FAM118s, just like in ThsA and Avs5, filament formation appears to be a prerequisite for catalytic activity (Fig. 7f,g). The dependence of activity on filament formation is emerging as a broader theme in biochemistry, particularly among metabolic enzymes, where filamentation is fairly frequently encountered and can stabilize active or inhibited enzyme conformations^65,66^. In the field of NAD-dependent enzymes, activation through filament formation has apparently independently evolved multiple times in TIR domain-containing NADases such as SARM1 (ref. ^67^), in the PARP-family ADP-ribosyltransferases tankyrases, which polymerize through an SAM domain^68^, and in at least three of the mentioned sirtuins.

While ThsA, Avs5 and FAM118s appear to share an analogous mechanism, important differences exist. For the first two, filament formation and, thus, NAD-processing activity are induced in response to specific signals produced upon phage infection^32,38^. In contrast, FAM118B forms filaments spontaneously in a concentration-dependent manner (Fig. 4a), suggesting that its activity is regulated primarily by expression levels, with filament assembly potentially creating a nonlinear, threshold-like response: low activity at sub-filament concentrations and higher activity as filaments form. This could create a concentration-dependent, filament-mediated mechanism for constraining aberrant activation of NAD-consuming activity. However, given the relatively high protein concentrations required for assembly, additional, as-yet-unknown mechanisms (possibly involving PTMs or interactions with other factors) may promote FAM118 filamentation under specific conditions such as infection. Moreover, as our results show a crucial interplay of the two FAM118 paralogs in catalytic activity (Fig. 7d–f), any mechanisms that prevent or facilitate their coassembly could provide a further regulatory layer.

In addition to modulating activity in some cases, filamentous self-assembly inherently increases the local concentration of individual protomers and can strengthen interactions with other repetitive molecules through cooperativity or avidity^42^. We do not know whether these mechanisms have a role in FAM118s but this remains a possibility. Increased local concentration afforded by filament formation could explain the oncogenic effects of *YAP1::FAM118B* chromosomal fusions observed in persons with cancer^69–71^. Similar to what has been proposed for other fusions involving filamentous proteins^72,73^, FAM118B filamentation could bring together multiple copies of fused YAP1, amplifying the binding capacity of this coactivator of proliferation genes and thereby promoting proliferation.

Filament formation can theoretically not only enhance individual, localized interaction events but also promote the formation of multivalent interaction networks. As a result, it is often the first step in the assembly of subcellular membraneless domains^42,74^. Consistent with this notion, we observed that both FAM118 paralogs, whether analyzed individually or together, can form distinct foci in cells when overexpressed past a certain threshold, with this behavior requiring an intact filament-forming interface (Fig. 6). These foci display high internal fluidity and likely contain correctly folded proteins, distinguishing them from mere aggregates (Fig. 6e,f). However, given the unnaturally high expression levels required, the foci may never form physiologically unless specific conditions or stimuli absent in our cellular assays promote their assembly; however, this remains speculative. This could resemble the behavior of immune-signaling players containing CARD or PYD domains (for instance, ASC), which tend to form ‘specks’ spontaneously in generic cell lines only at high concentrations but do so at endogenous levels upon infection in immune cells^75–79^. Lastly, FAM118B foci do not colocalize with the Cajal body marker coilin (Fig. 6c), indicating that the spontaneous clustering of the overexpressed protein is distinct from its previously proposed role in Cajal body assembly^48^.

In light of the recent discovery of FAM118B’s role in innate immunity^39^, sirtuins join a growing list of elements conserved between bacterial antiphage and eukaryotic innate immune systems^39^, such as cyclic GMP–AMP synthase, stimulator of interferon genes (STING) and TIR domain-containing proteins^67,80–82^. What also seems to be shared among these systems are certain mechanisms, one of which is filament formation, observed in the vertebrate inflammasomes^78,83–85^, in STING^86,87^ and TIR domain-containing proteins^88,89^ from both prokaryotes and eukaryotes, in bacterial sirtuins^32,38,90^ and now in FAM118s.

A further recurring theme that FAM118 apparently represent is the connection between immunity and NAD metabolism. In bacteria, TIR and sirtuin domains, both capable of processing NAD (by hydrolyzing it or transforming it into a secondary-messenger molecule), are integral to various antiphage systems. In eukaryotes, although the exact link between immune signaling and NAD levels remains unclear, a relation between NAD depletion and chronic inflammation has been observed and NAD precursors modulate immune reactions^91^. TIR domains related to those in bacterial antiphage systems are ubiquitous in eukaryotic innate immune receptors and adaptors and—although most of them might serve as catalytically inactive scaffolds—at least some, such as those in the mammalian adaptor SARM1 and plant TNL resistosomes, have NADase activity^67,88^. Given their homology to NAD-hydrolyzing bacterial sirtuins (Fig. 1a,b), we propose that FAM118s also function primarily as NADases, although other similar NAD-processing activities cannot be fully excluded. The fact that the NAD-processing activity of FAM118B:FAM118A, albeit much higher than that of either paralog alone, remains relatively modest may align with the intracellular, signaling function of FAM118s, in contrast to extracellular or cell-death-inducing roles of potent NADases, such as FoNADase1 (ref. ^62^) (Fig. 7d,e).

Overall, our findings uncover molecular mechanisms (filament formation and an FAM118B:FAM118A partnership in NAD processing) that likely contribute to a finely tuned system, with potential roles as a filamentous scaffold and an NAD degrader in immunity and other cellular processes. Future tag-free and endogenous-level validations will further consolidate these conclusions. From an evolutionary standpoint, our study underscores both deep homologies and striking mechanistic analogies between bacterial and eukaryotic immunity factors.

## Methods

### Phylogenetic analysis

For the sirtuin family tree, sequences of proteins containing different types of sirtuin domains were extracted using InterPro^92^ and National Center for Biotechnology Information (NCBI) resources^93,94^. For the FAM118B clade tree, orthologs of human FAM118B and FAM118A were identified using NCBI resources. In both cases, sirtuin domains were defined using the Pfam database^95^. The evolutionary history was inferred using the maximum-likelihood method and either the Whelan and Goldman model^96^ (sirtuin family tree) or the Jones–Taylor–Thornton (JTT) matrix-based model^97^ (FAM118-clade tree). The tree with the highest log likelihood (−30,365.33 for the sirtuin tree and −2,081.08 for the FAM118-clade tree) is presented. The initial trees for the heuristic search were obtained automatically by applying neighbor-joining (NJ) and BioNJ algorithms to a matrix of pairwise distances estimated with the JTT model and then selecting the topology with superior log-likelihood value. A discrete gamma distribution was used to model evolutionary rate differences among sites (five categories; +G, parameter = 3.2422 for sirtuin family tree or 0.9762 for FAM118-clade tree). For the sirtuin family analyses, the rate variation model allowed for some sites to be evolutionarily invariable ([+I], 0.25% sites). The final datasets contained a total of 399 (sirtuin family tree) or 152 (FAM118-clade tree) positions and the evolutionary analyses involved 86-aa or 30-aa sequences, respectively. Evolutionary analyses were conducted in MEGA X^98^.

### AF modeling

AF2 was accessed through ColabFold^99^ (version 1.5.5) run with default settings, including multiple-sequence alignment generation through MMseqs2 (many-against-many sequence searching)^100^. AF3 (ref. ^101^) was accessed through the AF server, again with default settings. All models were deposited in ModelArchive^102^ as described in ‘Data Availability’.

### Other bioinformatic analyses

To identify proteins that are most closely related to human FAM118s in NCBI resources at the sequence level to determine whether FAM118s form a distinct clade, we performed a basic local alignment search tool among proteins. The sequence of either human FAM118 paralog was used as query against the nonredundant protein sequence database with default settings except for ‘max target sequences’ set to 5,000. Only results with sequence coverage of at least 70% were considered.

Multiple-sequence alignments to probe the conservation of specific residues were performed using Clustal Omega with default settings^103^.

To map sequence conservation onto an AF3 structural model, we first generated multiple-sequence alignments with Clustal Omega comprising either (1) a representative 15 members of the FAM118 clade (eight FAM118Bs and seven FAM118As) with sequence identity ranging from 42% to 95% or (2) four FAM118s (FAM118A and FAM118B from *Homo* *sapiens* and *S*. *canicula*) and 11 related non-FAM118 SIR2_2 proteins that are part of the same higher-level clade in Extended Data Fig. 2. The conservation scores were calculated and mapped onto a structural model using ConSurf^104,105^ performed using a user-provided sequence alignment.

All structural superpositions and alignments, including root-mean-square-deviation (r.m.s.d.) calculations, were performed in PyMol.

### Plasmids for recombinant protein production

Nucleotide sequences encoding full-length human FAM118A (UniProt Q9NWS6) and FAM118B (UniProt Q9BPY3) were cloned into the pDEST-17 and pET-28a(+) plasmids, respectively, for the production of N-terminally hexahistidine-tagged (His-tagged) proteins used in structural studies and NAD cleavage assays.

Additionally, a nucleotide sequence corresponding to residues 24–334 of human FAM118B was cloned into a pET-28a(+) plasmid for the production of the C-terminally His-tagged FAM118B_24__–334_ protein used in structural studies and analytical SEC.

Point mutations were introduced using a standard site-directed mutagenesis PCR protocol.

### Recombinant protein production

An appropriate plasmid was transformed into Rosetta2 (DE3) *E*. *coli*. Bacteria were grown in either 2× yeast extract tryptone medium (for full-length FAM118A and FAM118B proteins) or Luria–Bertani Lennox medium (for FAM118B_24__–334_) supplemented with an appropriate antibiotic. An expression culture was inoculated with a saturated starter culture (1:100 dilution) grown overnight from a single colony from a fresh transformation plate. Large-scale expression cultures (1–2 L) were incubated at 37 °C, with shaking at 180 rpm, until an optical density at 600 nm of 0.6–0.8 was reached before cooling to room temperature. Expression was then induced with 0.25 mM or 0.5 mM IPTG for full-length proteins or FAM118B_24__–334_, respectively. The induced cultures were incubated at 18 °C and 180 rpm for a further ~20–24 h before harvesting by centrifugation at 4,500*g* for 30 min. Cell pellets were collected and stored at −20 °C until protein purification.

### Protein purification

Recombinant full-length FAM118A and FAM118B used for structural studies and NAD cleavage assays were purified using a procedure consisting of Ni^2+^ affinity chromatography followed by SEC. Cell pellets were resuspended in lysis buffer (20 mM HEPES pH 7.5, 500 mM NaCl, 0.5 mM TCEP, 20 mM imidazole, 10 μg ml^−1^ DNAse I, 100 μg ml^−1^ lysozyme and 1× cOmplete EDTA-free protease inhibitor) and lysed by sonication before clarification by centrifugation at 18,000*g* for 30 min. The soluble fractions of cell lysates were incubated with Ni-NTA agarose (Cytiva) preequilibrated in wash buffer (20 mM HEPES pH 7.5, 500 mM NaCl, 0.5 mM TCEP and 20 mM imidazole). Bound proteins were washed using wash buffer and eluted using elution buffer (20 mM HEPES pH 7.5, 500 mM NaCl, 0.5 mM TCEP and 200 mM imidazole). Protein-containing fractions were concentrated down to a volume of 5 ml and injected onto a 16/600 HiLoad Superdex 200 prep-grade column (Cytiva) preequilibrated in SEC buffer (20 mM HEPES pH 7.5, 150 mM NaCl and 0.5 mM TCEP). The protein was eluted from the column using SEC buffer.

FAM118B_24__–334_ for structural studies and analytical SEC experiments was purified using a procedure comprising Ni^2+^ affinity and ion-exchange chromatography the cell pellet was resuspended in 25 mM Tris pH 8.8, 500 mM NaCl and 0.5 mM TCEP. Following sonication, the crude lysate was separated into the pellet and supernatant fractions by centrifugation (12,000*g* for 30 min at 10 °C). The supernatant was loaded onto a 5-ml HisTrap high-performance (HP) column (Cytiva) and the column was washed with 25 mM Tris pH 8.8, 500 mM NaCl and 0.5 mM TCEP first without and then with 50 mM imidazole, followed by protein elution with 250 mM imidazole. The eluted protein was then diluted ten times with water, loaded onto a 5-ml HiTrap Q HP column (Cytiva), equilibrated in 25 mM Tris pH 8.8 and 0.5 mM TCEP and eluted using a salt gradient from 50 to 1,000 mM NaCl in the same buffer. The final protein from the main ion-exchange peak was in an approximate buffer composition of 25 mM Tris pH 8.8, 250 mM NaCl and 0.5 mM TCEP.

All protein purification steps were performed at 4 °C, with protein purity at each stage assessed by SDS–PAGE. Final purified protein fractions were pooled, concentrated using a centrifugal concentrator, frozen in liquid nitrogen and stored in a −80 °C freezer.

### FAM118B negative-stain analysis

A thin film of carbon on top of a copper 200-mesh grid (C101/100, TAAB) was glow-discharged for 25 s at 15 mA of current (Pelco, easiGlow) before applying 3 μl of purified human FAM118B directly onto the grid. After 1 min of incubation at ambient temperature, the sample was absorbed by touching a side of the grid with a wedge of filter paper (Whatman, 1001-090). Facing down, the grid was rapidly dropped on top of 20 μl of 2% uranyl acetate solution dispensed onto the surface of a parafilm, where it was incubated for 10 s. The excess stain was blotted away until a thin film remained and the grid was air-dried for 5 min. The images were collected at a nominal magnification of ×50,000 and a pixel size of 2.3 Å on a 200-kV EM instrument, JEM-2100Plus (Jeol) (The Dunn School of Pathology, University of Oxford).

### FAM118B cryoEM sample preparation

A purified recombinant human FAM118B was diluted from thawed material to 0.1 mg ml^−1^ (3.5 μM) in 12.5 mM Tris pH 7.5, 150 mM NaCl and 0.5 mM TCEP buffer. Then, 3.5 μl was applied to the Quantifoil 1.2/1.3 Cu 300-mesh grids pretreated in Harrick plasma in vacuum for 2 min at high power. A Leica GP2 was equilibrated to 100% humidity at 18 °C before the sample was backblotted for 1.5 s and plunge-frozen in liquid ethane. The grids were stored in liquid nitrogen until the EM imaging.

### FAM118B cryoEM data collection and processing

A total of 11,630 raw cryoEM images were collected in a super-resolution mode at a physical pixel size of 0.83 Å on a 300-kV Titan Krios microscope (Thermo Fisher Scientific) with an integrated K3 camera and an energy filter (20-eV slits were used). The images were acquired at a defocus range of −1.6 μm to −2.6 μm and a sum dose per video of 43.7 e^−^ per Å^2^. The data were collected in the EM facility COSMIC (University of Oxford).

The entire dataset was preprocessed on the fly using cryoSPARC^106^ live (COSMIC, University of Oxford) when 40-frame videos were aligned using default settings of patch motion correction and the contrast transfer function (CTF) values were estimated using the Patch CTF. Particle picking was carried out using the blob picker with a blob diameter from 100 to 200 Å, resulting in 1,958,302 selected particles. Initially, all particles were extracted with a smaller box size of 320 pixels and a binning factor of 4 and were subjected to two-dimensional (2D) classification. The images representing the FAM188B filaments were retained, while classes most obviously presenting as bright ice contaminants were manually deselected. The ab initio models constrained to five classes were calculated from 1,540,301 particles, which were used as reference models for a round of heterogeneous refinement. A single good three-dimensional (3D) class reminiscent of a filament reconstructed from 531,673 images (34.5%) was reextracted with a box size of 600 pixels (498 Å) and subjected to a homogeneous refinement to 6.4-Å resolution. A resulting map showed discernible core of a filament made up from five repeating units; however, lateral contacts between the core and adjacent units were also found. With such a 3D map reconstructed from 2D image sampling of a linear segment of filaments, its branching points or occasional aggregates (evident from subsequent 2D classification but also in our initial negative-stain EM images), a second round of ab initio reconstruction limited to five classes was attempted. This step was followed by heterogeneous refinement forcing hard classification so that each particle was assigned to only one class at each iteration. Four meaningful 3D classes were obtained representing clearly defined filaments short (2–4-mers) and long (5–7-mer). The longest filament was the only class (111,156 images) where no lateral contacts between a linear core and adjacent subunits could be observed, which was then further subjected to a nonuniform focused refinement using a binary mask (soft padding of 50 pixels and dilation radius of 1 pixel) encompassing the length of a filament fragment comprising five protomers. To better resolve the structure of the head-to-tail interfaces and the NAD-binding pocket, all four classes of filaments were combined (368,283 particles) before carrying out a nonuniform focused refinement within a binary mask (soft padding of 60 pixels and dilation radius of 2 pixels) around a core part encompassing two full protomers and fragments of flanking protomers. The final cryoEM maps of filament fragments composed of five and two protomers reconstructed at an overall resolution (gold-standard Fourier shell correlation (FSC) = 0.143) of 5 Å and 4.1 Å, respectively, were used for structural modeling of a full-length FAM118B filament.

### FAM118B cryoEM structural modeling

Individual chains of AF3-predicted structures of human FAM118B (Q9BPY3-F1) were first rigid-body fitted in the map of a full-length FAM118B filament fragment encompassing two protomers (4.1 Å) using ChimeraX^107^. Disordered N-terminal (1–27) and C-terminal (327–351) tails, present in the AF3 model, were found outside of the cryoEM density and were removed using Coot^108^. Each of the two molecules of FAM118B was then flexibly fitted using molecular dynamics flexible fitting implemented in ISOLDE^109^ as a ChimeraX plugin. Torsional and distance self-restraints were applied during simulations. Occasionally, large bulky side chains were manually repositioned especially around the NAD-binding pocket and at the interfaces between the protomers. The process was repeated for fitting five individual molecules of FAM118B models into the map of the full-length FAM118B filament fragment encompassing five protomers.

### FAM118B_24__–334_ negative-stain analysis

A total of 4 µl of FAM118B_24__–334_ at 3 µM (diluted from thawed protein stock in 25 mM Tris pH 8.8, 250 mM NaCl and 0.5 mM TCEP) was applied to glow-discharged 300-mesh Formvar grids (EMS) for 60 s at room temperature. Following sample application, the grids were manually blotted to remove excess liquid. Subsequently, 20 µl of uranyl acetate was applied three times for 30 s each. The grids were then air-dried before imaging. The negative-stained grids were first screened using a Tecnai T12 microscope operated at 120 kV.

### FAM118B_24__–334_ cryoEM sample preparation

A total of 3 µl of FAM118B_24__–334_ at 30 µM (diluted from thawed protein stock in 25 mM Tris pH 8.8, 250 mM NaCl and 0.5 mM TCEP) was applied to negatively glow-discharged 200-mesh Lacey grids (EMS). Grids were blotted with ash-free Whatman grade 540 filter paper in a Vitrobot Mark IV (Thermo Fisher Scientific) for 4 s at 4 °C and 95–100% humidity and then vitrified in liquid ethane. The sample quality and distribution were initially assessed using a Glacios microscope (Thermo Fisher Scientific) equipped with a Falcon 4 direct electron detector (Thermo Fisher Scientific) (NanoImaging Core Facility, Institut Pasteur).

### FAM118B_24__–334_ cryoEM data collection and processing

A preliminary dataset of 100 images was collected at the NanoImaging Core Facility of the Pasteur Institute using a Glacios microscope, operated at 200 kV and equipped with a BioQuantum Energy Filter (Gatan) with a 10-eV slit width. Videos were recorded using a Falcon direct electron detector in super-resolution mode at a magnification of ×105,000, corresponding to a pixel size of 0.83 Å. A dose rate of 15 e^−^ per s per physical pixel resulted in a total electron dose of 40 e^−^ per Å^2^, applied over 40 frames. Data collection was performed using EPU software with defocus values ranging from −1 to −3 µm.

Video stacks were processed in cryoSPARC^106^. Super-resolution videos were frame-aligned, motion-corrected, gain-normalized, dose-weighted and binned twice using the patch motion correction module. CTF parameters were estimated with the patch CTF module in cryoSPARC. Micrographs exhibiting ice contamination, ethane contamination or poor CTF fit resolution (below 6 Å) were discarded.

Initial particle picking was performed using a circular blob picker with dimensions of 40–250 Å. Following an initial round of 2D classification, ab initio reconstruction and heterogeneous refinement, a template-based picker was applied to improve particle selection. A second round of 2D classification, ab initio reconstruction and heterogeneous refinement was conducted. Finally, 50,985 particles were selected for nonuniform refinement, resulting in the final reconstruction map.

The final dataset of 19,798 videos was collected at the European Synchrotron Radiation Facility (ESRF) Grenoble (CM01 beamline)^110^ using a Titan Krios microscope (Thermo Fisher Scientific) operated at 300 kV using a K3 camera (Gatan) in super-resolution acquisition mode and equipped with a quantum LS Energy Filter (Gatan) with a 20-eV slit width at a magnification of ×105,000, corresponding to a pixel size of 0.839 Å. A dose rate of 18.9 e^−^ per s per physical pixel resulted in a total electron dose of 40.18 e^−^ per Å^2^, applied over 40 frames. Data collection was performed using EPU software with defocus values ranging from −0.8 to −2.8 µm. Video stacks were processed in cryoSPARC. Super-resolution videos were frame-aligned, motion-corrected, gain-normalized, dose-weighted and binned twice using the patch motion correction module. CTF parameters were estimated with the patch CTF module in cryoSPARC. Micrographs exhibiting ice contamination, ethane contamination or poor CTF fit resolution (below 6 Å) were discarded.

Leveraging the reconstruction map obtained from the small dataset, we directly performed template picking for particle selection. A round of 2D classification, ab initio reconstruction and heterogeneous refinement was applied as described above and illustrated in Extended Data Fig. 7. The final map obtained from nonuniform refinement was derived from a filament composed of six protomers and reached a medium resolution of approximately 8.7 Å, as estimated by the FSC = 0.143 criterion, using the PDBe FSC server. To improve the resolution of individual protomers and get a better view of the interfaces between subunits, a local refinement was performed focusing on a subset of three protomers. This refinement yielded a final map with an improved resolution of around 4.8 Å, as determined by the FSC = 0.143 criterion using the PDBe FSC server. A local resolution map was subsequently calculated in PHENIX^111,112^ (from half-maps), confirming that the refined region achieved resolutions ranging approximately from 3.6 to 7 Å.

### FAM118B_24__–334_ cryoEM structural modeling

The AF model of human FAM118B, residues 24–334, was fitted into the density of the focused map of the filament fragment composed of three protomers using ChimeraX^107^. Multiple rounds of refinement in Coot^108^ and PHENIX^111,112^ were applied, resulting in a model with a cross-correlation of around 0.6 and mean *Q* score of 0.2.

### Analytical SEC

Analytical SEC analysis was performed using a 10/300 GL Superdex 100 Increase column (Cytiva) in 50 mM Tris pH 8, 200 mM NaCl and 0.5 mM TCEP pumped at 1 ml min^−1^. WT or mutant FAM118B_24__–334_ protein samples diluted to the indicated concentrations with the same buffer were prepared in a total volume of 515 µl and injected onto the column using a 500-µl loop. Then, 1-ml fractions were collected during the run and 7 µl of each from the relevant elution volume (EV) range was analyzed by SDS–PAGE.

The column was calibrated using a collection of molecules of various MW values, which eluted at indicated EVs: blue dextran (MW, 2,000 kDa; EV, 8.7 ml), ferritin (MW, 440 kDa; EV, 9.75 ml), immunoglobulin G (MW, 160 kDa; EV, 11.4 ml), BSA (MW, 66 kDa; EV, 13.3 ml), ovalbumin (MW, 44 kDa; EV, 14.6 ml), carbonic anhydrase (MW, 29 kDa; EV, 16.2 ml) and ribonuclease A (MW, 13.7 kDa; EV, 17.9 ml). A linear regression between EV and log_10_MW was determined with Excel and applied to predict the theoretical EV values for various multimerization states of FAM118B_24__–334_.

### ITC

ITC experiments were performed at 25 °C using a PEAQ-ITC apparatus (Malvern instruments). FAM118B_24__–334_-L32R, present in the syringe at 500 µM, was titrated into FAM118B_24__–334_-F122R, present in the cell at 50 µM. A total of 19 injections were carried out, each of 0.5 ul, with 1 s for injection and 150 s between injections. The stirring speed was set to 750 rpm. Both proteins were exchanged into 1× PBS buffer before the experiment through ten rounds of dilution and concentration in a centrifugal concentrator. The ITC data were analyzed with the MicroCal PEAQ-ITC analysis software (version 1.41) with subtraction of injection heat of buffer into buffer, L32R mutant into buffer and buffer into F122R mutant. The *K*_D_ could not be reliably estimated because of a high-affinity interaction. Attempts to perform ITC with lower concentrations of both proteins were unsuccessful because of a low signal-to-noise ratio.

### DLS

DLS experiments were performed using Zetasizer Nano S (Malvern Instruments) at 20 °C. FAM118B_24__–334_ WT was diluted to 10 µM in PBS buffer (137 mM NaCl, 2.7 mM KCl, 10 mM Na_2_HPO_4_ and 1.8 mM KH_2_PO4, pH 7.4). The data were analyzed with Zetasizer software.

### Plasmids for cell biology experiments

The full-length native DNA sequence of human FAM118B (UniProt Q9BPY3) was synthesized and cloned either into (1) the pEGFP-C1 plasmid, in which the gene encoding the EGFP tag was mutated to render it monomeric (A206K substitution, the final tag designated as mEGFP), or (2) the pmCherry-C1 plasmid.

The full-length native DNA sequences of human FAM118A (UniProt Q9NWS6) and FAM118B were also cloned into pDEST-YFP-N1 (ref. ^113^) or pDEST-mCherry-N1 (ref. ^114^) using Gateway recombination (Invitrogen).

Site-directed mutagenesis was performed using a standard site-directed PCR-based approach.

### Cell culture

U2OS cells (from I. Ahel’s laboratory^115^, originally ATCC-HTB-96) were maintained in DMEM supplemented with 10% FBS (Sigma) and penicillin–streptomycin (100 U per ml, Gibco) and cultured in a humidified atmosphere at 37 °C with 5% CO_2_. U2OS were plated in eight-well glass-bottom chamber slides (Ibidi or ZellContact) 24 h before transfection of plasmids using TransIT-LT1 (Mirus Bio) or X-tremeGENE HP (Sigma) transfection reagents according to the manufacturer’s instructions.

### Fluorescence confocal imaging

For Fig. 6a,b, U2OS cells transiently expressing fluorescently labeled FAM118A and FAM118B were incubated with 1.25 µg ml^−1^ Hoescht 33342 for 30 min, before replacing the medium with imaging medium (phenol-red-free Leibovitz’s L-15 medium (Life Technologies) supplemented with 20% FBS, penicillin (100 μg ml^−1^) and streptomycin (100 U per ml)). Cells were imaged on an Olympus IX-83 inverted microscope equipped with a Yokogawa SoRa super-resolution spinning-disk head, a UPlanAop ×60 (1.5 numerical aperture (NA)) oil-immersion objective lens and a Prime BSI scientific complementary metal–oxide–semiconductor camera. The fluorescence of Hoechst and YFP was excited with 405-nm and 488-nm solid state lasers, respectively, and fluorescence detection was achieved with bandpass filters adapted to the fluorophore emission spectra. For Fig. 6c,h, U2OS cells transiently expressing mCherry-tagged or mEGFP-tagged proteins were imaged on an LSM 880 Zeiss confocal microscope equipped with a water-immersion C-Apo ×40 (1.2 NA) lens. mEGFP and mCherry were excited using 488-nm and 561-nm lasers, respectively, and fluorescence detection was collected at 500–550 nm for mEGFP and 580–650 nm for mCherry using GaAsP detectors. The pixel size was set to 70 nm.

### FCCS and PCH

PCH and FCCS experiments were performed on an LSM 880 Zeiss confocal microscope equipped with a water-immersion C-Apo ×40 (1.2 NA) lens. mEGFP and mCherry were excited using 488-nm and 561-nm lasers, respectively. Fluorescence fluctuations were collected at 500–550 nm for mEGFP and 580–650 nm for mCherry, both on the GaAsP detector used in photon-counting mode. Laser powers were adjusted to minimize photobleaching and acquisitions lasted 20–30 s to reduce noise on the autocorrelation and cross-correlation curves and PCHs. Cells were maintained at 37 °C with a heating chamber. PCHs were calculated from the raw fluorescence fluctuation data using time bins of 100 µs and fitted with a one-specie model assuming a 3D Gaussian detection volume using the Zen Black software^116^. The values obtained for the number of diffusing oligomers in the confocal volume and their average brightness were corrected for diffusion as previously described^117^. The concentrations of the labeled proteins were calculated assuming a confocal volume of ~1 fl (ref. ^118^). For FCCS analysis, the autocorrelation and cross-correlation curves were calculated from raw photon traces after detrending for slow fluctuations using the Fluctuation Analyzer 4G software^119^. The autocorrelation and cross-correlation curves were fitted using one-specie simple diffusion models assuming a shape factor equal to 6. To correct for unavoidable nonidealities in the acquisition setup, we used positive controls, corresponding to cells expressing mEGFP linked to mCherry, and negative controls, corresponding to cells coexpressing free mEGFP and mCherry. The bleedthrough between the mEGFP and mCherry channels was estimated from the negative controls and subtracted from the other FCCS acquisitions. Differences in autocorrelation amplitudes and incomplete cross-correlation because of partial overlap between the confocal volumes in the mEGFP and mCherry were corrected using the positive controls as previously described^118^.

### NADase assay

NADase activity was assessed using a plate-reader-based fluorescent ε-NAD cleavage assay. Reactions (15-μl final volume) comprising purified FAM118B and/or FAM118A protein (at 5 µM each), reaction buffer (20 mM HEPES pH 7.5, 150 mM NaCl and 0.5 mM TCEP) and 0.5 mM ε-NAD were prepared in PCR tubes and transferred to 384-well black polystyrene nonbinding clear-bottom assay plates. In the positive control reaction, porcine brain NADase (Sigma-Aldrich, N9879) at the final concentration of 1 mg ml^−1^ was used instead of FAM118 protein. Fluorescence recordings (300-nm excitation, 410-nm emission) were measured using the SpectraMax M5 plate reader (Molecular Devices) in kinetic mode every 30 s for 180 min.

### Cellular NAD levels

Cellular NAD levels were assayed using the NAD/NADH colorimetric assay kit (Abcam, ab65348) following the manufacturer’s protocol. First, 2 days before the assay, 6 × 10^5^ 293T cells (ATCC-CRL-3216) were seeded into a six-well plate (Corning Costar 3516). The next day, cells were treated with either 2 nM FK866 (NAMPT inhibitor) or DMSO or transfected with 2 µg of plasmid DNA per well of pDEST-YFP, pDEST-YFP-FAM118A WT, pDEST-YFP-FAM118A-N148A, pDEST-YFP-FAM118A-F109R, pDEST-YFP-FAM118B WT, pDEST-YFP-FAM118B-N168A or pDEST-YFP-FAM118-F122R using 20 µl of Polifect (Qiagen, 301105) following the manufacturer’s instructions. In summary, Polyfect–DNA complexes were assembled in 75 µl of serum-free medium (1× DMEM GlutaMAX; Gibco, 31966-021) and incubated at room temperature for 10 min. Thereafter, the Polyfect–DNA mixture was added dropwise onto 2.5 ml of complete medium per well supplemented with 10% FBS (Sigma, 9665) and 1% penicillin–streptomycin (Gibco, 15140-122). Then, 24 h later, cells were harvested, washed with 1 ml of PBS (Sigma, D8537) and trypsinized in 0.5 ml of TrypLE Express (Gibco, 12604-013). Cells were resuspended in 2 ml of complete medium with an average count of 1 × 10^6^ cells per ml. The 6 × 10^5^ cells were sampled, briefly pelleted at 4 °C and washed twice with ice-cold PBS to remove residual trypsin. Then, 400 ul of extraction buffer II at ambient temperature was used for cell lysis by two repeated freeze–thaw cycles on dry ice. Cells were vortexed for 10 s and centrifuged at 16,873*g* (maximum speed) for 5 min at 4 °C to remove insoluble material (leaving behind a transparent loose pellet). The supernatant was passed through a 10-kDa spin column (Abcam, ab93349) at 16,873*g* for 1 h at 4 °C to remove endogenous NAD/NADH-consuming enzymes. The flowthrough from each sample was split into two to allow independent measurements of total cellular NAD and NADH (NADt) or NADH only. To measure only NADH, NAD was depleted by heating the samples to 60 °C for 30 min followed by cooling down on ice for 5 min to avoid denaturing of the cycling enzyme in the next step. Next, the NADH standard was freshly prepared from a DMSO stock by diluting 1:100 (10 pmol µl^−1^) in extraction buffer II to a final concentration of 20–100 pmol per well. Next, 50 μl of sample in triplicates (NADt and NADH only) and NADH standard in duplicates was dispensed in a 96-well flat, clear polystyrene microplate (Corning, CLS3370). Then, 100 μl of reaction master mix (cycling buffer I and NAD cycling enzyme mix) was added to each well before mixing on a plate shaker for 5 min at ambient temperature. Lastly, 10 μl of developer solution II was added to each sample and standard before incubating on a plate shaker for 15 min at room temperature. The absorbance at 450 nm was measured on a plate reader SpectraMax 5/SoftMax Pro v7 (Molecular Devices) over the course of 4 h with 10-min intervals at 21 °C. NADt and NADH levels were calculated from absorbance values using an NADH standard curve. The cellular levels of NAD (pmol per well) were calculated by subtracting NADH-only values from NADt values. The NAD levels were normalized to the total protein content (pmol µg^−1^ protein) of each sample determined by the Bradford Protein Assay (Bio-Rad) and the BSA (Thermo Scientific, 23209) standard curve. Statistical analyses were performed in GraphPad Prism (version 10.5).

### Western blotting

To monitor protein levels as shown in Supplementary Figure 3, lysates were boiled in 1× NuPAGE LDS sample buffer (Invitrogen) with 50 mM DTT (Sigma-Aldrich). Thereafter, proteins were subjected to NuPAGE Novex 4–12% Bis–Tris gel (Invitrogen), followed by semidry transfer onto a 0.2-µm nitrocellulose membrane (Bio-Rad) using the Trans-Blot Turbo transfer system (Bio-Rad) and blocking in 5% nonfat dried milk in 1× PBS buffer with 0.1% Tween-20 (PBST) for 1 h at room temperature. Goat anti-GFP antibody was diluted in 5% nonfat dried milk in PBST (Abcam, ab5450; batch GR3402450-2, stock concentration 0.5 mg ml^−1^, dilution 1:5,000) and used overnight at 4 °C followed by horseradish-peroxidase-conjugated rabbit anti-goat secondary antibody incubation (Dako, P0160; stock concentration 0.65 mg ml^−1^, dilution 1:7,500) for 45 min at room temperature. Pierce enhanced chemiluminescence western blotting substrate (Thermo Fisher Scientific) was added onto the membrane for 5 min, followed by visualization on Hyperfilm ECL films (Cytiva).

### Statistics and reproducibility

No statistical methods were used to determine sample sizes. Data distribution was assumed to be normal but this was not formally tested.

The sample size for cryoEM data was arbitrarily chosen depending on instrument availability and the number of particles required for standard structural reconstruction, ensuring good final statistics.

Sample sizes (numbers of cells) for cellular experiment were arbitrarily chosen to be around 30 (the precise values are indicated for each experiment in figure legends) to span the variability within the data, providing a reliable mean estimate. For qualitative assessment of protein localization, we analyzed *n* = 15 cells at low and *n* = 15 cells at high expression levels to produce representative images.

The numbers of measurements in quantitative NAD-processing and NAD-level assays were arbitrarily chosen to be *n* = 3 or *n* = 4, respectively, to allow an estimate of s.d. or s.e.m. To determine the significance of differences in cellular NAD levels (Fig. 7g), a one-way analysis of variance (ANOVA) and Tukey’s post hoc test for multiple comparisons or unpaired one-tailed *t*-test for a binary comparison was applied as indicated in figure legend.

All quantitative and qualitative assays were repeated independently at least *n* = 2 times with similar conclusions, as indicated in figure legends. All attempts at replication for the experiments included in the manuscript were successful.

No data were excluded except for some particles being disregarded during cryoEM analysis as per best practice in cryoEM data analysis, as explained in detail.

No randomization or blinding was performed.

### Reporting summary

Further information on research design is available in the Nature Portfolio Reporting Summary linked to this article.

## Online content

Any methods, additional references, Nature Portfolio reporting summaries, source data, extended data, supplementary information, acknowledgements, peer review information; details of author contributions and competing interests; and statements of data and code availability are available at 10.1038/s41594-025-01715-1.

## Supplementary information

Supplementary Information Supplementary Figs. 1–3 and source images for Supplementary Fig. 3.

Reporting Summary

## Source data

Source Data Fig. 4 Uncropped gels.

Source Data Fig. 4 Measurement source data for DLS and ITC.

Source Data Fig. 5 Measurement source data for graphs.

Source Data Fig. 6 Measurement source data for graphs.

Source Data Fig. 7 Measurement and statistical source data for graphs.

Source Data Extended Data Fig. 2 List of proteins with species and accession codes used in the phylogenetic tree.

Source Data Extended Data Fig. 3 List of proteins with species and accession codes used in the phylogenetic tree.

## Data Availability

CryoEM maps were deposited to the EM Data Bank under accession codes EMD-53497, EMD-53488, EMD-53555 and EMD-53556. CryoEM model coordinates were deposited to the PDB under accession codes 9R0P, 9R0S, and 9R3E. AF models were deposited to ModelArchive under identifiers ma-qjds4 (1×FAM118B, AF3), ma-lomyz (1×FAM118B+NAD), ma-tcwfj (1×FAM118A, AF3), ma-hg5eg (2×FAM118B, AF3), ma-u0dco (FAM118A:FAM118B, AF3), ma-td690 (1×FAM118B, AF2), ma-vowtz (1×FAM118A, AF2), ma-yp5tm (2×FAM118B, AF2), ma-1gx7g (2×FAM118A, AF2) and ma-az2ng (FAM118A:FAM118B, AF2). Source data are provided with this paper. All other data supporting the findings of this study are available from the corresponding authors on reasonable request.
