# Supplementary material for: Filament formation and NAD processing by noncanonical human FAM118 sirtuins
Source: Nat Struct Mol Biol. 2025 Nov 17;32(12):2526–41. doi: 10.1038/s41594-025-01715-1 (PMC12700824; doi:10.1038/s41594-025-01715-1)
Supplement: Supplementary file 1 — Supplementary Figs. 1–3 and source images for Supplementary Fig. 3. [file 41594_2025_1715_MOESM1_ESM.pdf]

nature structural & molecular biology

Supplementary information

https://doi.org/10.1038/s41594-025-01715-1

Filament formation and NAD processing by noncanonical human FAM118 sirtuins

In the format provided by the authors and unedited

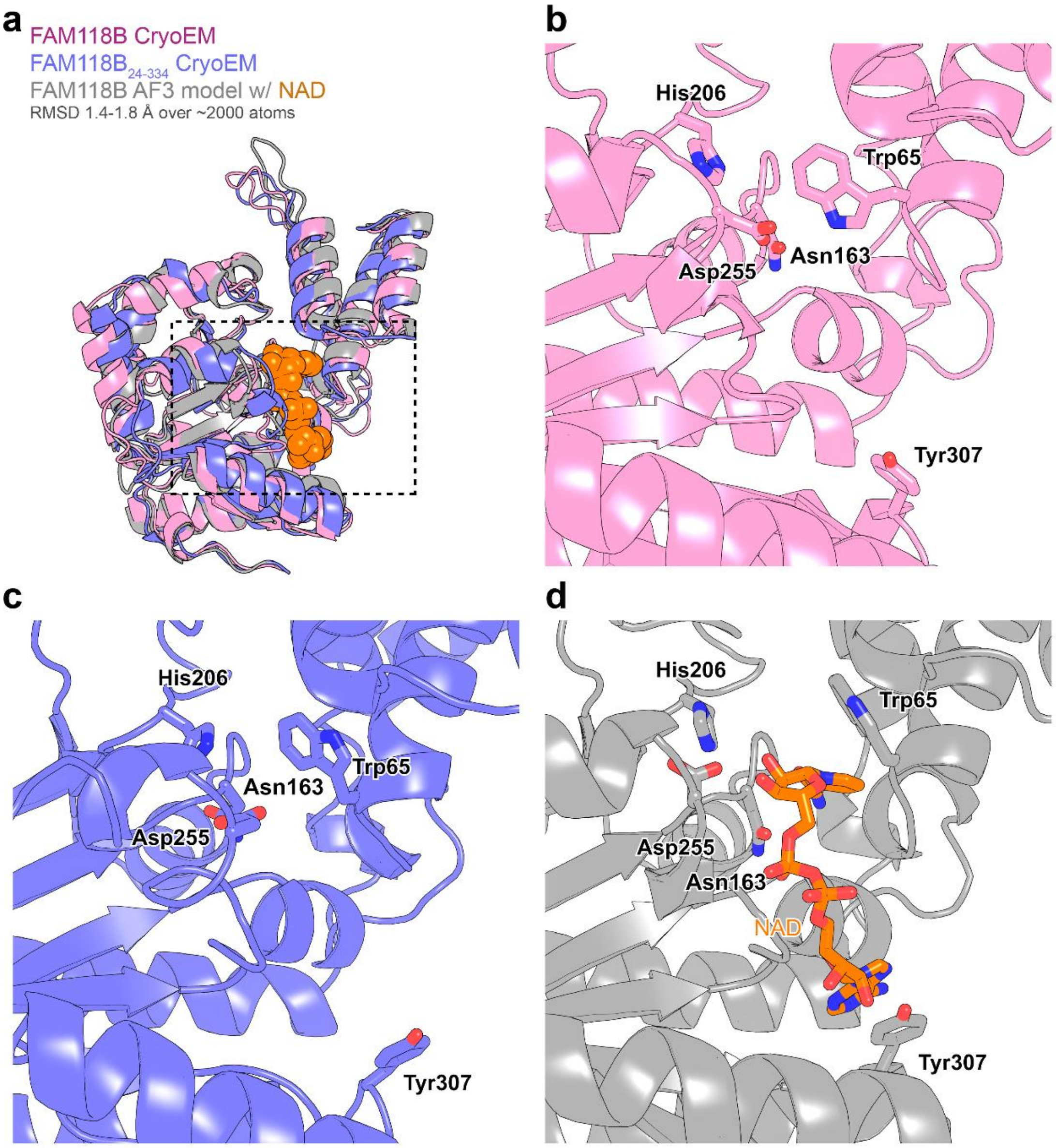

Supplementary Fig. 1: Structural comparison of the cryoEM-derived models of NAD-Free FAM118B protomer and the AlphaFold3 model of NAD-bound FAM118B

a, A representative protomer model derived from the full-length (pink) or 24-334 (blue) FAM118B filament cryoEM reconstruction superposed with an AlphaFold3 (AF3) model of FAM118B (residues 28-325) bound to nicotinamide dinucleotide (NAD) (grey with NAD in orange). The root-mean-square-deviation (RMSD) value range for pairwise structural alignments of the three models over the indicated number of well-aligned atoms is provided. The region zoomed-in in panels b, c, and d is indicated with a dotted line. **b**, Zoomed-in NAD-binding site in the cryoEM-derived model of NAD-free full-length FAM118B with selected residues indicated as sticks and labelled. **c**, Zoomed-in NAD-binding site in the cryoEM-derived model of NAD-free FAM118B$_{24\text{-}334}$ with selected residues indicated as sticks and labelled. **d**, Zoomed-in NAD-binding site of NAD-bound FAM118B AF3 model with NAD (in orange except for blue for nitrogen atoms and red for oxygen atoms) and selected residues indicated as sticks and labelled.

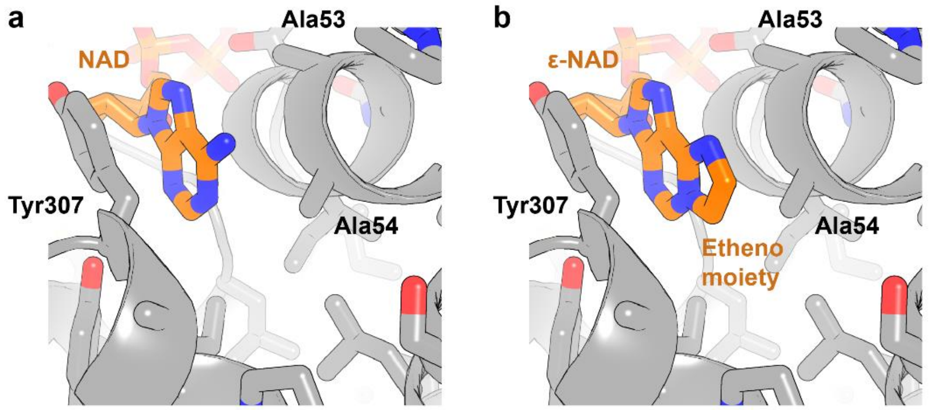

Supplementary Fig. 2: Structural modelling suggests ε-NAD can be accommodated in the FAM118B active site

a, Close-up view of the apparent adenosine-binding pocket in the AlphaFold3 (AF3) model of NAD-bound human FAM118B (from **Extended Data Fig. 1c**).

b, Close-up view on the same model as in a, but with adenosine of NAD replaced with etheno-adenosine. The replacement was performed by structurally aligning the etheno-adenosine part of ε-NAD derived from the PDB entry 2X0J onto the adenosine part of normal NAD using the pair_fit function of PyMol.

In both panels, the amino-acid sidechains in the direct vicinity of (etheno-)adenosine are labelled.

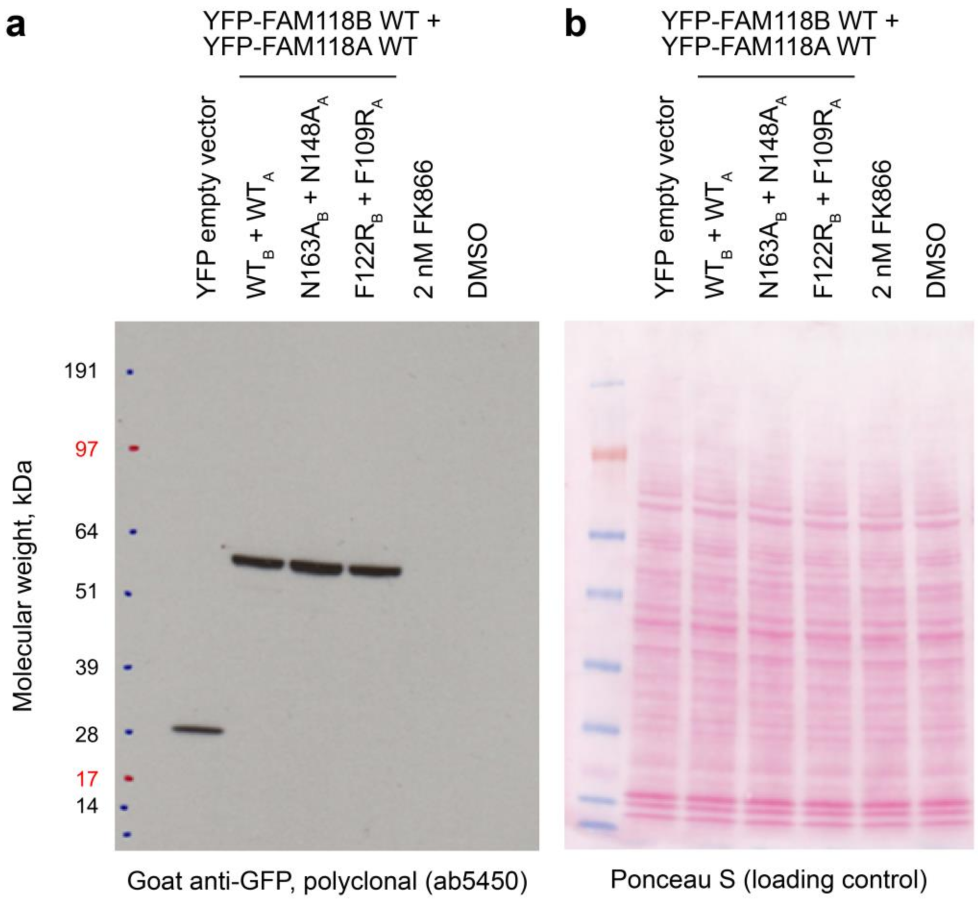

Supplementary Fig. 3: Protein level control of human 293T cells transiently transfected with vectors encoding YFP-tagged FAM118 proteins

This result belongs to the experiment shown in **Fig. 8g**.

a, Protein levels of free YFP (from empty vector) or YFP-tagged FAM11B and YFP-tagged FAM118A (from two simultaneously transfected vectors) monitored with immunoblotting using the indicated anti-GFP antibody. “A” or “B” in subscript refers to YFP-FAM118A or YFP-FAM118B, respectively.

b, Total protein on the same membrane is stained with Ponceau S as a loading control.

Uncropped blots for this panel are provided on the next page.

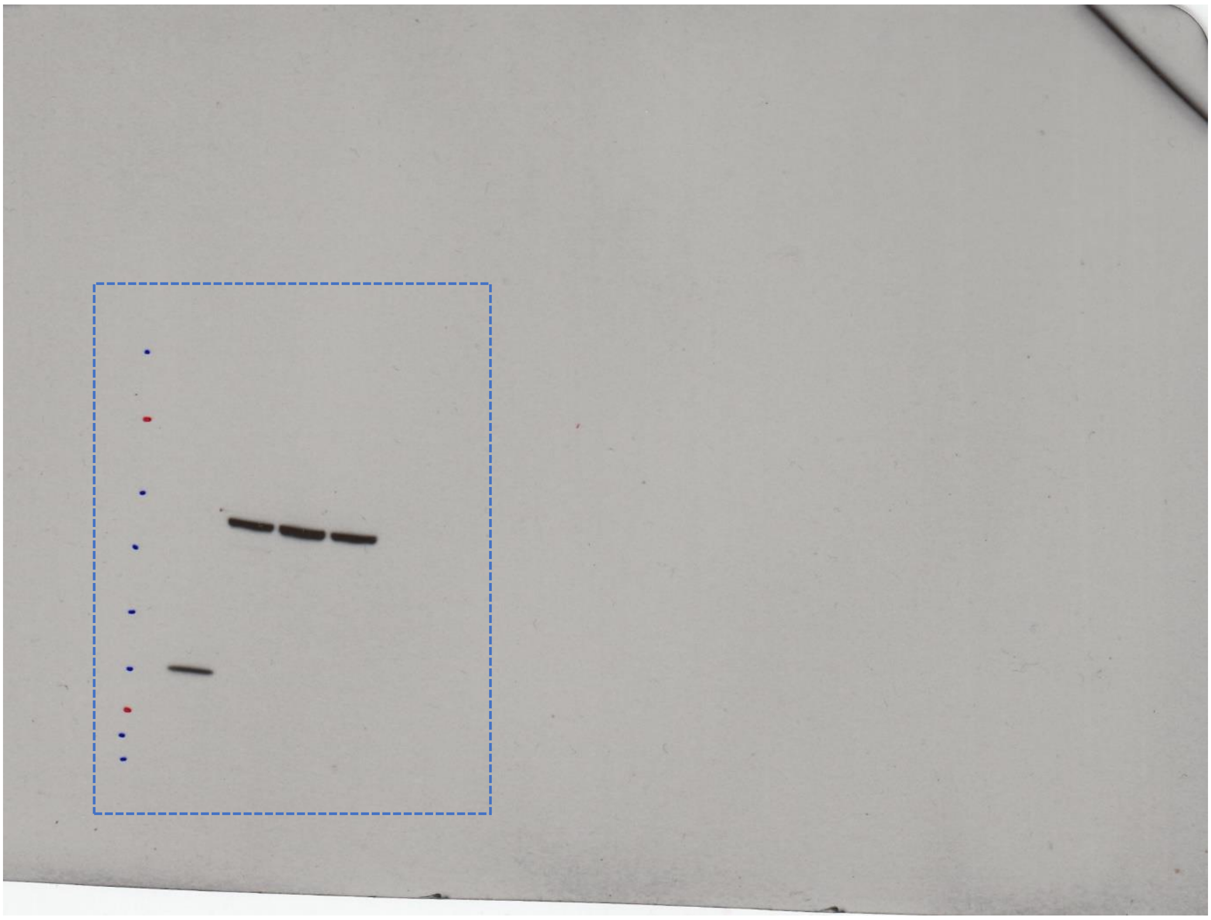

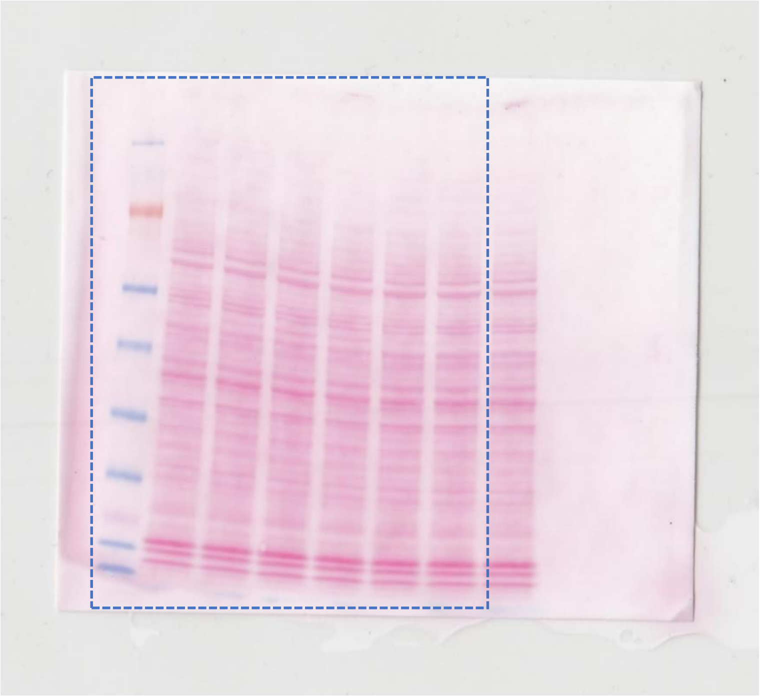

Uncropped blots for Supplementary Figure 3

The areas presented in Supplementary Figure 3 are approximately marked with dashed rectangles.

Top, a photo of a Hyperfilm ECL film showing an exposure of a membrane probed with goat anti-GFP primary antibody (ab5450) and HRP-conjugated rabbit anti-goat secondary antibody (Dako P0160) and visualised using Pierce ECL Western Blotting Substrate solution.

Bottom, a photo of the same membrane as above stained with Ponceau S protein stain and briefly destained with water.
