# Supplementary material for: Filament formation and NAD processing by noncanonical human FAM118 sirtuins
Source: Nat Struct Mol Biol. 2025 Nov 17;32(12):2526–41. doi: 10.1038/s41594-025-01715-1 (PMC12700824; doi:10.1038/s41594-025-01715-1)
Supplement: Supplementary file 2 — Reporting Summary [file 41594_2025_1715_MOESM2_ESM.pdf]

nature portfolio

Corresponding author(s): Huet, S; Ahel, I; Suskiewicz, MJ

Last updated by author(s): 15/10/25

Reporting Summary

Nature Portfolio wishes to improve the reproducibility of the work that we publish. This form provides structure for consistency and transparency in reporting. For further information on Nature Portfolio policies, see our Editorial Policies and the Editorial Policy Checklist.

Statistics

For all statistical analyses, confirm that the following items are present in the figure legend, table legend, main text, or Methods section.

n/a	Confirmed	
☐	☒	The exact sample size (*n*) for each experimental group/condition, given as a discrete number and unit of measurement
☐	☒	A statement on whether measurements were taken from distinct samples or whether the same sample was measured repeatedly
☐	☒	The statistical test(s) used AND whether they are one- or two-sided<br>*Only common tests should be described solely by name; describe more complex techniques in the Methods section.*
☒	☐	A description of all covariates tested
☒	☐	A description of any assumptions or corrections, such as tests of normality and adjustment for multiple comparisons
☐	☒	A full description of the statistical parameters including central tendency (e.g. means) or other basic estimates (e.g. regression coefficient) AND variation (e.g. standard deviation) or associated estimates of uncertainty (e.g. confidence intervals)
☐	☒	For null hypothesis testing, the test statistic (e.g. *F*, *t*, *r*) with confidence intervals, effect sizes, degrees of freedom and *P* value noted<br>*Give P values as exact values whenever suitable.*
☒	☐	For Bayesian analysis, information on the choice of priors and Markov chain Monte Carlo settings
☒	☐	For hierarchical and complex designs, identification of the appropriate level for tests and full reporting of outcomes
☒	☐	Estimates of effect sizes (e.g. Cohen's *d*, Pearson's *r*), indicating how they were calculated

Our web collection on statistics for biologists contains articles on many of the points above.

Software and code

Policy information about availability of computer code

Data collection	AKTA Unicorn 7 for protein purification and analytical SEC; Zetasizer for DLS; MicroCal PEAQ-ITC Control Software v1.41 for ITC; Softmax Pro 5 for NADase assay; ThermoFisher EPU software for automated cryoEM data acquisition
Data analysis	AKTA Unicorn 7 for analytical SEC; Zetasizer for DLS; MicroCal PEAQ-ITC Analysis Software v1.41 for ITC; Softmax Pro 5 and MS Excel for NAD-processing and NAD levels assay; CryoSPARC 4.5.3 for CryoEM data analysis; Zen Black 2.3 for photon counting histogram analyses; Fluctuations Analyzer 4G for analysing fluorescence cross-correlation spectroscopy data; MEGA X 12 version 12.0.14 250820 for phylogenetic/evolutionary analyses; AlphaFold2 accessed through ColabFold v1.5.5 for structure prediction; AlphaFold3 (version AlphaFold-beta-20231127) accessed through AlphaFold Server for structure prediction; Clustal Omega 1.2.4 for sequence alignments; PyMol 2.6.0a0 Open-Source for structural alignments and structural figures; ChimerX with ISOLDE plugin for cryoEM electron density analysis and model fitting

For manuscripts utilizing custom algorithms or software that are central to the research but not yet described in published literature, software must be made available to editors and reviewers. We strongly encourage code deposition in a community repository (e.g. GitHub). See the Nature Portfolio guidelines for submitting code & software for further information.

Data

Policy information about availability of data

All manuscripts must include a data availability statement. This statement should provide the following information, where applicable:

- Accession codes, unique identifiers, or web links for publicly available datasets
- A description of any restrictions on data availability
- For clinical datasets or third party data, please ensure that the statement adheres to our policy

CryoEM maps were deposited to the Electron Microscopy Data Bank (EMDB) under accession codes EMD-53497, EMD-53488, EMD-53555, and EMD-53556. CryoEM model coordinates were deposited to the Protein Data Bank (PDB) under the accession codes 9R0P, 9R0S, and 9R3E. AlphaFold models were deposited to ModelArchive under identifiers ma-qjds4 (1xFAM118B, AF3), ma-lomyz (1xFAM118B+NAD), ma-tcwfj (1xFAM118A, AF3), ma-hg5eg (2xFAM118B, AF3), ma-u0dco (FAM118A:FAM118B, AF3), ma-td690 (1xFAM118B, AF2), ma-vowtz (1xFAM118A, AF2), ma-yp5tm (2xFAM118B, AF2), ma-1gx7g (2xFAM118A, AF2), ma-az2ng (FAM118A:FAM118B, AF2).
Source data have been provided in Source Data (see the last page of Supplementary Information for their inventory). All other data supporting the findings of this study are available from the corresponding authors on reasonable request.

Research involving human participants, their data, or biological material

Policy information about studies with human participants or human data. See also policy information about sex, gender (identity/presentation), and sexual orientation and race, ethnicity and racism.

Reporting on sex and gender	*Use the terms sex (biological attribute) and gender (shaped by social and cultural circumstances) carefully in order to avoid confusing both terms. Indicate if findings apply to only one sex or gender; describe whether sex and gender were considered in study design; whether sex and/or gender was determined based on self-reporting or assigned and methods used.<br>Provide in the source data disaggregated sex and gender data, where this information has been collected, and if consent has been obtained for sharing of individual-level data; provide overall numbers in this Reporting Summary. Please state if this information has not been collected.<br>Report sex- and gender-based analyses where performed, justify reasons for lack of sex- and gender-based analysis.*
Reporting on race, ethnicity, or other socially relevant groupings	*Please specify the socially constructed or socially relevant categorization variable(s) used in your manuscript and explain why they were used. Please note that such variables should not be used as proxies for other socially constructed/relevant variables (for example, race or ethnicity should not be used as a proxy for socioeconomic status).<br>Provide clear definitions of the relevant terms used, how they were provided (by the participants/respondents, the researchers, or third parties), and the method(s) used to classify people into the different categories (e.g. self-report, census or administrative data, social media data, etc.)<br>Please provide details about how you controlled for confounding variables in your analyses.*
Population characteristics	*Describe the covariate-relevant population characteristics of the human research participants (e.g. age, genotypic information, past and current diagnosis and treatment categories). If you filled out the behavioural & social sciences study design questions and have nothing to add here, write "See above."*
Recruitment	*Describe how participants were recruited. Outline any potential self-selection bias or other biases that may be present and how these are likely to impact results.*
Ethics oversight	*Identify the organization(s) that approved the study protocol.*

Note that full information on the approval of the study protocol must also be provided in the manuscript.

Field-specific reporting

Please select the one below that is the best fit for your research. If you are not sure, read the appropriate sections before making your selection.

☒ Life sciences ☐ Behavioural & social sciences ☐ Ecological, evolutionary & environmental sciences

For a reference copy of the document with all sections, see nature.com/documents/nr-reporting-summary-flat.pdf

Life sciences study design

All studies must disclose on these points even when the disclosure is negative.

Sample size	No statistical methods were used to determine sample sizes. Data distribution was assumed to be normal but this was not formally tested.<br>Sample size for cryo-EM data was arbitrarily chosen depending on instrument availability and the number of particles required for standard structural reconstruction, ensuring good final statistics.<br>Sample sizes (numbers of cells) for cellular experiment were arbitrarily chosen to be around 30 (the precise values are indicated for each experiment in figure legends) in order to span the variability within the data, providing a reliable mean estimate. For qualitative assessment of protein localization, we analysed n = 15 cells at low and n = 15 cells at high expression levels to produce representative images.<br>The numbers of measurements in quantitative NAD-processing and NAD levels assays were arbitrarily chosen to be n = 3 or n = 4, respectively, to allow an estimate of standard deviation or standard error of the mean.

Data exclusions	No data were excluded except for some particles being disregarded during cryoEM analysis as per best practice in cryoEM data analysis, and as explained in detail in Methods and Extended Data Figures.
Replication	All quantitative and qualitative assays were repeated independently at least n = 2 times with similar conclusions, as indicated in figure legends. All attempts at replication for the experiments included in the manuscript were successful.
Randomization	No randomisation
Blinding	No blinding

Reporting for specific materials, systems and methods

We require information from authors about some types of materials, experimental systems and methods used in many studies. Here, indicate whether each material, system or method listed is relevant to your study. If you are not sure if a list item applies to your research, read the appropriate section before selecting a response.

Materials & experimental systems

n/a	Involved in the study
☐	☒ Antibodies
☐	☒ Eukaryotic cell lines
☒	☐ Palaeontology and archaeology
☒	☐ Animals and other organisms
☒	☐ Clinical data
☒	☐ Dual use research of concern
☒	☐ Plants

Methods

n/a	Involved in the study
☒	☐ ChIP-seq
☒	☐ Flow cytometry
☒	☐ MRI-based neuroimaging

Antibodies

Antibodies used	Anti-GFP (Abcam, ab5450), rabbit anti-goat (Dako, P0160)
Validation	Anti-GFP antibody (ab5450) is a goat polyclonal antibody and was validated by the manufacturer for use in EM, ICC/IF, IHC - Wmt, IHC-FoFr, IHC-P, IP, WB. Rabbit anti-goat (P0160) is a secondary rabbit polyclonal antibody and is recommended by the manufacturer for immunohistochemistry, ELISA, and immunoblotting. According to the manufacturer, it is particularly sensitive but can react with immunoglobulins from other species (which is not a limitation for our use).

Eukaryotic cell lines

Policy information about cell lines and Sex and Gender in Research

Cell line source(s)	Experiments were performed in the U2OS cell line (from one of our laboratories: Ivan Ahel, see Gibbs-Seymour, et al., Mol Cell, 2016; original ATCC-HTB-96) and in the 293T cell line (purchased from ATCC-CRL-3216).
Authentication	U2OS and 293T cell lines were identified by morphology but were not authenticated
Mycoplasma contamination	All cells were routinely tested for mycoplasma contamination using commercial mycoplasma testing kit and used only when negative
Commonly misidentified lines (See ICLAC register)	No such lines were used

Plants

Seed stocks	*Report on the source of all seed stocks or other plant material used. If applicable, state the seed stock centre and catalogue number. If plant specimens were collected from the field, describe the collection location, date and sampling procedures.*
Novel plant genotypes	*Describe the methods by which all novel plant genotypes were produced. This includes those generated by transgenic approaches, gene editing, chemical/radiation-based mutagenesis and hybridization. For transgenic lines, describe the transformation method, the number of independent lines analyzed and the generation upon which experiments were performed. For gene-edited lines, describe the editor used, the endogenous sequence targeted for editing, the targeting guide RNA sequence (if applicable) and how the editor was applied.*
Authentication	*Describe any authentication procedures for each seed stock used or novel genotype generated. Describe any experiments used to assess the effect of a mutation and, where applicable, how potential secondary effects (e.g. second site T-DNA insertions, mosiacism, off-target gene editing) were examined.*
