# Supplementary figures and images for: Filament formation and NAD processing by noncanonical human FAM118 sirtuins
Source: Nat Struct Mol Biol. 2025 Nov 17;32(12):2526–41. doi: 10.1038/s41594-025-01715-1 (PMC12700824; doi:10.1038/s41594-025-01715-1)

FAM118B WT

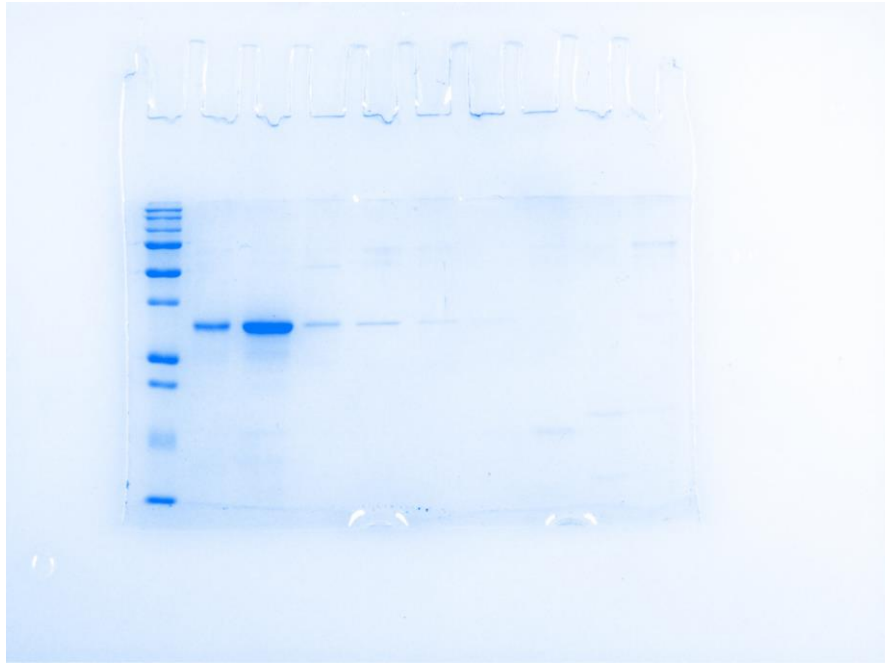

FAM118B L32R

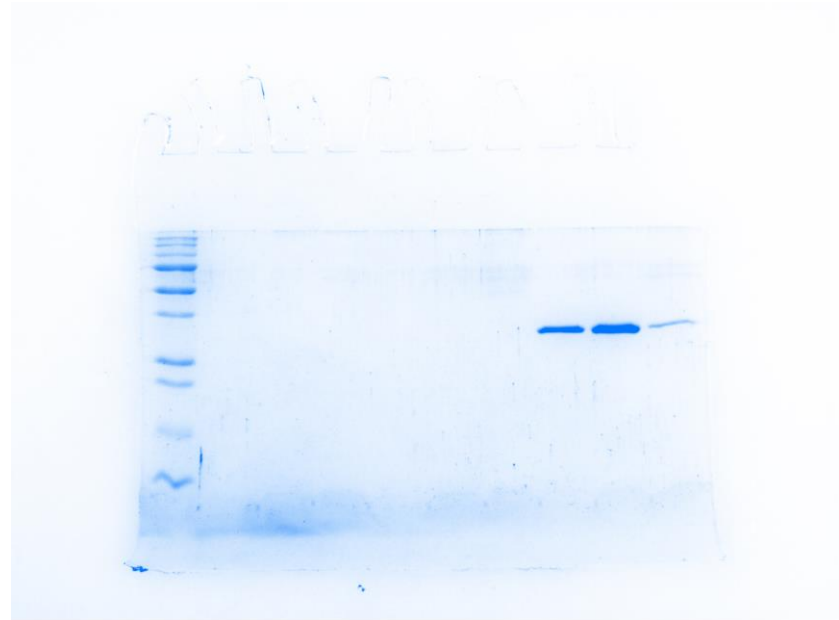

FAM118B F122R

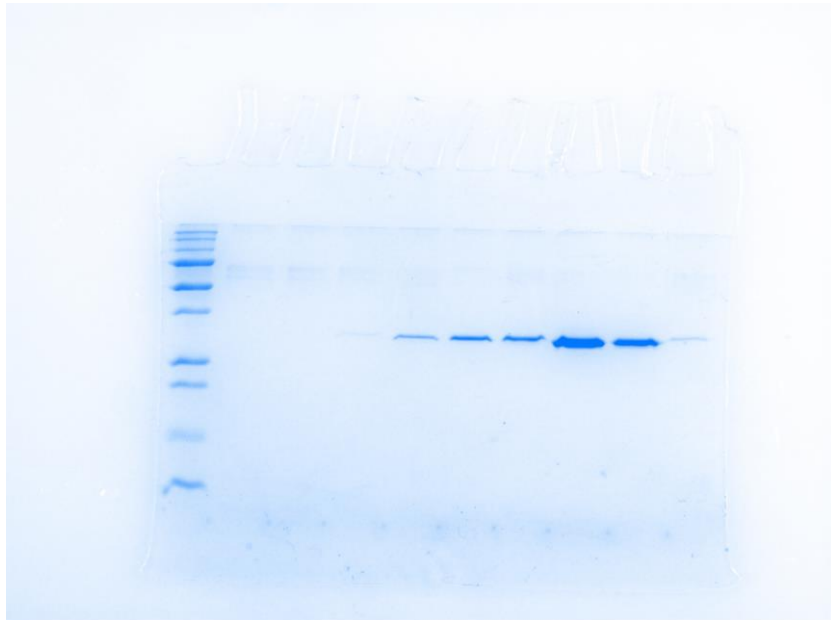

FAM118B F122R + 32R

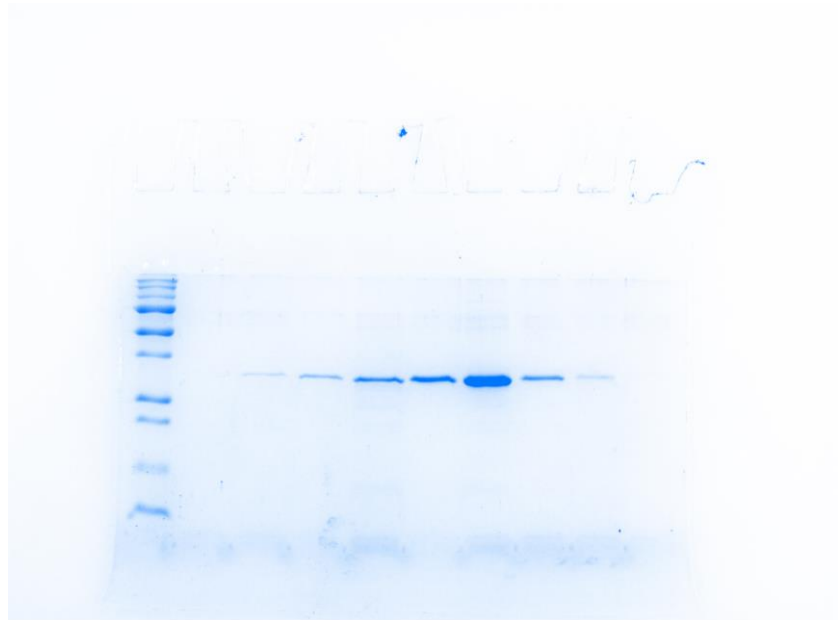

Supplement: Supplementary file 3 — Uncropped gels. [file 41594_2025_1715_MOESM3_ESM.pdf]
